# Supplementary material for: Programmable melanoma-targeted radio-immunotherapy via fusogenic liposomes functionalized with multivariate-gated aptamer assemblies
Source: Nat Commun. 2024 Jun 12;15:5035. doi: 10.1038/s41467-024-49482-9 (PMC11169524; doi:10.1038/s41467-024-49482-9)
Supplement: Supplementary file 1 — Supplementary Information [file 41467_2024_49482_MOESM1_ESM.pdf]

Supplementary Information:

Programmable melanoma-targeted radio-immunotherapy via fusogenic liposomes functionalized with multivariate-gated aptamer assemblies

Xijiao Ren,[1†] Rui Xue,[2†] Yan Luo, [3†] Shuang Wang,[2] Xinyue Ge,[2] Xuemei Yao,[2] Liqi Li,[4] Junxia Min,[5] Menghuan Li, [2*] Zhong Luo,[1,2*] Fudi Wang [5,6*]

[1] Key Laboratory of Biorheological Science and Technology, Ministry of Education, Chongqing University, Chongqing 400044, P. R. China.

[2] School of Life Science, Chongqing University, Chongqing 400044, P. R. China.

[3] Radiation Oncology Center, Chongqing University Cancer Hospital, Chongqing 400030, P.R. China

[4] Department of General Surgery, Xinqiao Hospital, Army Medical University, Chongqing 400037, P. R. China.

[5] The Second Affiliated Hospital, The First Affiliated Hospital School of Public Health Institute of Translational Medicine State Key Laboratory of Experimental Hematology, Zhejiang University School of Medicine, Hangzhou 310058, China.

[6] The First Affiliated Hospital Basic Medical Sciences, School of Public Health Hengyang Medical School University of South China, Hengyang 421001, P. R. China.

[†] Xijiao Ren, Rui Xue and Yan Luo contributed equally to this work.

Email:

Fudi Wang: fwang@zju.edu.cn;

Zhong Luo: luozhong918@cqu.edu.cn;

Menghuan Li: menghuanli@cqu.edu.cn

Name	Sequence (5'-3' or N-C terminus)
aptATP-Chol	ACCTGGGGGAGTATTGCGGAGGAAGGTAAGATTTTTT-Chol
CpG ODN	TCCATGACGTTCCTGACGTT
CpG ODN-Cy5	TCCATGACGTTCCTGACGTT-Cy5
eCpG	GCAATACTCCTCCATGACGTTCCTGACGTT
eCpG-Cy5	GCAATACTCCTCCATGACGTTCCTGACGTT-Cy5
Mutational CpG ODN	TCCATGAAATTCCTGACGTT
Mutational CpG ODN-Cy5	TCCATGAAATTCCTGACGTT-Cy5
Mutational eCpG	GCAATACTCCTCCATGAAATTCCTGACGTT
Mutational eCpG-Cy5	GCAATACTCCTCCATGAAATTCCTGACGTT-Cy5
Closed eCpG	CTGCAACTCCTCCATGACGTTCCTGACGTT
Closed eCpG-Cy5	CTGCAACTCCTCCATGACGTTCCTGACGTT-Cy5
PNA	K-tctta-PLGVRG-ttcct-K
aptPD-L1-Chol	CCAACGGTCCATGTGTTCAGACGAGACGAACTTATGCGTATAC<br>ATTGTCACTCCCCGCCATTTTTT-Chol
FAM-aptPD-L1-Chol	FAM-<br>CCAACGGTCCATGTGTTCAGACGAGACGAACTTATGCGTATAC<br>ATTGTCACTCCCCGCCATTTTTT-Chol

Supplementary Table 1. Details related to the nucleic acid-based materials.

Sequences for aptamers and peptide nucleic acids used in this paper.

Number	Name	Reactivity	Cat	dilution	Source
**1**	PC7-anti-CD45 antibody	Mouse	E-AB-F1136G	1:50	elabscience
**2**	APC-anti-CD45 antibody	Mouse	E-AB-F1136E	1:50	elabscience
**3**	APC-anti-CD3 antibody	Mouse	E-AB-F1013E	1:50	elabscience
**4**	APC-anti-CD11c antibody	Mouse	E-AB-F0991E	1:50	elabscience
**5**	PE-anti-CD8a antibody	Mouse	E-AB-F1104D	1:50	elabscience
**6**	PE-anti-CD4 antibody	Mouse	E-AB-F1097D	1:50	elabscience
**7**	PE-anti-CD86 antibody	Mouse	E-AB-F0994D	1:50	elabscience
**8**	FITC-anti-IFN-γ antibody	Mouse	E-AB-F1101C	1:50	elabscience
**9**	FITC-anti-CD44 antibody	Mouse	E-AB-F1100C	1:50	elabscience
**10**	PE-anti-MHC-II antibody	Mouse	E-AB-F0990D	1:50	elabscience
**11**	FITC-anti-CD4 antibody	Mouse	E-AB-F1097C	1:50	elabscience
**12**	PE-anti-CD11c antibody	Mouse	E-AB-F0991D	1:50	elabscience
**13**	PE-anti-GR1 antibody	Mouse	E-AB-F1120D	1:50	elabscience
**14**	FITC-anti-CD11b antibody	Mouse	E-AB-F1081C	1:50	elabscience
**15**	FITC-anti-CD80 antibody	Mouse	E-AB-F0992C	1:50	elabscience
**16**	APC-anti-CD25 antibody	Mouse	E-AB-F1102E	1:50	elabscience
**17**	APC-anti-CD62L antibody	Mouse	E-AB-F1011E	1:50	elabscience
**18**	APC-anti-CD80 antibody	Mouse	E-AB-F0992E	1:50	elabscience
**19**	FITC-anti-CTLA-4 antibody	Mouse	ab24935	1:100	Abcam
**20**	anti-VEGFA antibody	Mouse	19003-1-AP	1:500	proteintech
**21**	anti-HIF-1α antibody	Mouse	66730-1-Ig	1:500	proteintech
**22**	anti-pERK1/2 antibody	Mouse	28733-1-AP	1:500	proteintech
**23**	anti-PARP1 antibody	Mouse	66520-1-Ig	1:500	proteintech
**24**	anti-γH2AX antibody	Mouse	10856-1-AP	1:500	proteintech
**25**	anti-β-Tubulin antibody	Mouse	M30109S	1:1000	Abmart
**26**	anti-Calretinin antibody	Mouse	ab92341	1:200	Abcam
**27**	anti-HMGB1 antibody	Mouse	ab79823	1:200	Abcam
**28**	anti-PD-L1 antibody	Mouse	ab213480	1:200	Abcam
**29**	FITC-anti-PD-L1 antibody	Mouse	E-AB-F1132C	1:50	elabscience

Supplementary Table 2. Details related to antibodies. The information for antibodies used in this paper.

Number	Name	Validation information
1	PC7-anti-CD45 antibody	https://www.elabscience.cn/p-pe_cyanine5_anti_mouse_cd45_antibody_30_f11_-172697.html
2	APC-anti-CD45 antibody	https://www.elabscience.cn/p-apc_anti_mouse_cd45_antibody_30_f11_-172695.html
3	APC-anti-CD3 antibody	https://www.elabscience.cn/p-apc_anti_mouse_cd3_antibody_17a2_-133030.html
4	APC-anti-CD11c antibody	https://www.elabscience.cn/p-apc_anti_mouse_cd11c_antibody_n418_-132974.html
5	PE-anti-CD8a antibody	https://www.elabscience.cn/p-pe_anti_mouse_cd8a_antibody_53_6.7_-133053.html
6	PE-anti-CD4 antibody	https://u2.elabscience.cn/p-pe_anti_mouse_cd4_antibody_gk1.5_-133045.html
7	PE-anti-CD86 antibody	https://u2.elabscience.cn/p-pe_anti_mouse_cd86_antibody_gl_1_-172246.html
8	FITC-anti-IFN-γ antibody	https://www.elabscience.cn/p-fitc_anti_mouse_ifn_gamma_antibody_xmg1.2_-172590.html
9	FITC-anti-CD44 antibody	https://www.elabscience.cn/p-fitc_anti_human_mouse_cd44_antibody_im7_-176555.html
10	PE-anti-MHC-II antibody	https://www.elabscience.cn/p-pe_anti_mouse_mhc_ii_i_a_i_e_antibody_m5_114_-132965.html
11	FITC-anti-CD4 antibody	https://u2.elabscience.cn/p-fitc_anti_mouse_cd4_antibody_gk1.5_-133044.html
12	PE-anti-CD11c antibody	https://www.elabscience.cn/p-pe_anti_mouse_cd11c_antibody_n418-e_ab_f0991d
13	PE-anti-GR1 antibody	https://www.elabscience.cn/p-pe_anti_mouse_ly_6g_ly_6c_gr_1_antibody_rb6_8c5_-150590.html
14	FITC-anti-CD11b antibody	https://www.elabscience.cn/p-fitc_anti_mouse_human_cd11b_antibody_m1_70_-133068.html
15	FITC-anti-CD80 antibody	https://u2.elabscience.cn/p-fitc_anti_mouse_cd80_antibody_16_10a1_-172238.html
16	APC-anti-CD25 antibody	https://www.elabscience.cn/p-apc_anti_mouse_cd25_antibody_pc_61.5.3_-134626.html
17	APC-anti-CD62L antibody	https://www.elabscience.cn/p-apc_anti_mouse_cd62l_antibody_mel14_-172281.html
18	APC-anti-CD80 antibody	https://u2.elabscience.cn/p-apc_anti_mouse_cd80_antibody_16_10a1_-134498.html
19	FITC-anti-CTLA-4 antibody	https://www.abcam.cn/products/primary-antibodies/fitc-ctla4-antibody-1b8-ab24935.html
20	anti-VEGFA antibody	https://www.ptgcn.com/products/VEGFA-Antibody-19003-1-AP.htm
21	anti-HIF-1α antibody	https://www.ptgcn.com/products/HIF1a-Antibody-66730-1-Ig.htm
22	anti-pERK1/2 antibody	https://www.ptgcn.com/products/ERK1-2-phospho-Thr202-Tyr204-Antibody-28733-1-AP.htm
23	anti-PARP1 antibody	https://www.ptgcn.com/products/PARP1-Antibody-66520-1-Ig.htm
24	anti-γH2AX antibody	https://www.ptgcn.com/products/H2AFX-Antibody-10856-1-AP.htm
25	anti-β-Tubulin antibody	http://www.ab-mart.com.cn/page.aspx?node=%2059%20&id=%20992
26	anti-Calretinin antibody	https://www.abcam.cn/products/primary-antibodies/calretinin-antibody-ep1798-ab92341.html
27	anti-HMGB1 antibody	https://www.abcam.cn/products/primary-antibodies/hmgb1-antibody-epr3507-ab79823.html

**28**	anti-PD-L1 antibody	https://www.abcam.cn/products/primary-antibodies/pd-l1-antibody-epr20529-ab213480.html
**29**	FITC-anti-PD-L1 antibody	https://www.elabscience.cn/p-fitc_anti_mouse_cd274_pd_l1_antibody_10f_9g2-e_ab_f1132c

Supplementary Table 3. Antibody validation. The validation information for antibodies used in this paper.

Liposome type	Hydrodynamic size (nm)	Polydispersity index (PDI)	Zeta potential (mV)	Encapsulation efficiency (%)	Relative encapsulation amount (%)
Lip	121.96 ± 1.27	0.1163 ± 0.0025	38.16 ± 1.84	/	/
Lip@AUR	125.76 ± 1.74	0.1207 ± 0.0025	29.97 ± 1.29	93.07 ± 4.65	5.25 ± 0.26
Lip@AUR-ACP	128.55 ± 1.92	0.1230 ± 0.0026	1.01 ± 0.46	89.89 ± 3.79	5.07 ± 0.21
Lip@AUR-ACP-aptPD-L1	129.83 ± 2.92	0.1247 ± 0.0031	-10.71 ± 1.22	88.29 ± 3.16	4.98 ± 0.18

Supplementary Table 4. Summarization of the physicochemical properties of the intermediates and final product of the liposomes. The Summarization of Figure 2, Supplementary Figure 2 and Supplementary Figure 3.

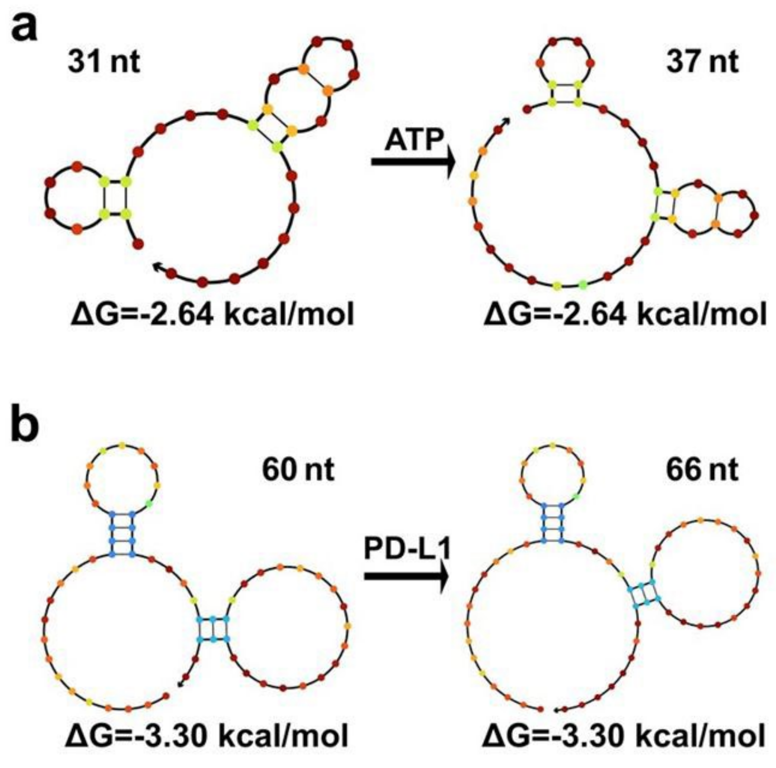

Supplementary Figure 1. Molecular simulation results for key aptamer components. NUPACK analysis of (a) aptATP and (b) aptPD-L1 after molecular engineering.

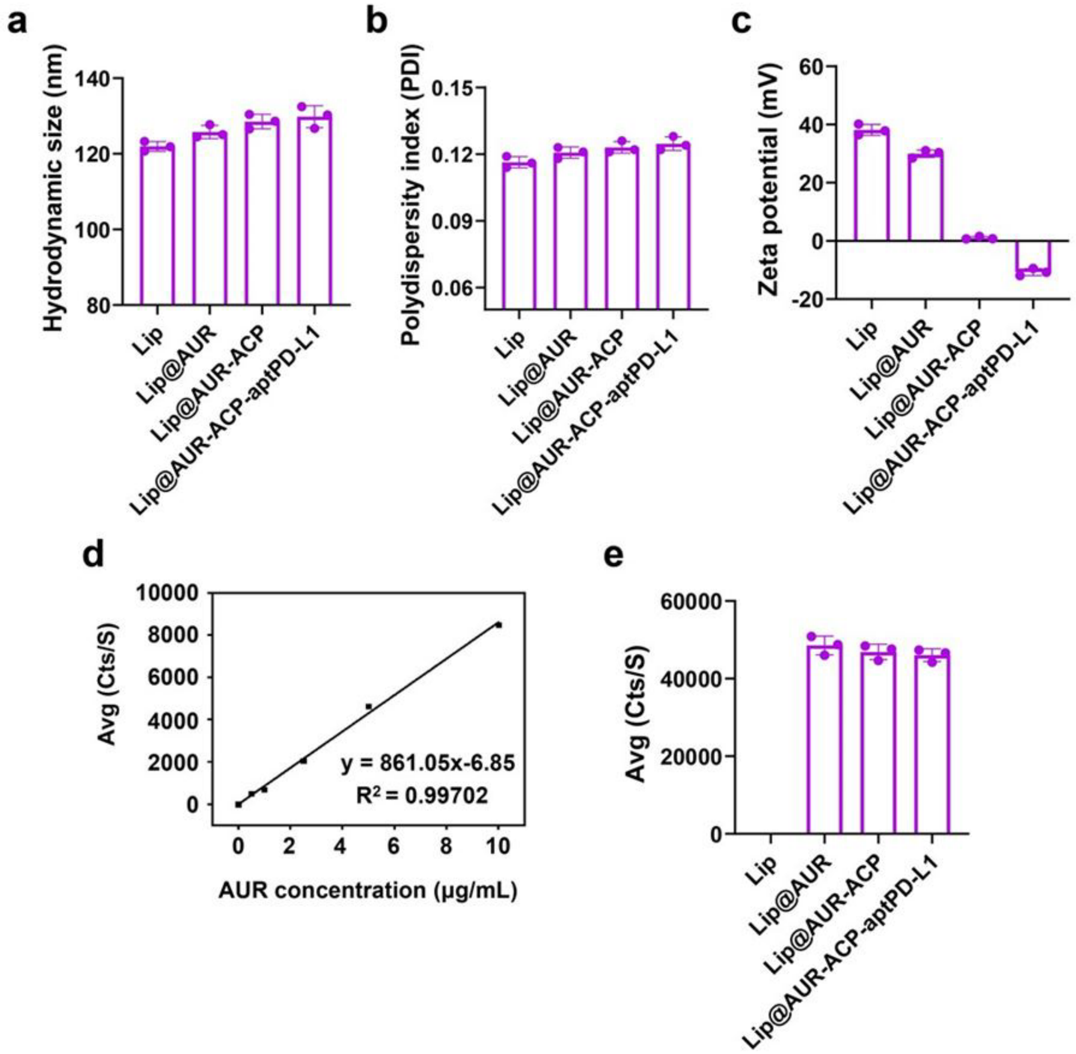

Supplementary Figure 2. Physical and chemical properties of the liposome sample series. (a-c) The hydrodynamic size, polydispersity index and zeta potential of liposomes by DLS. (d) The standard curve with AUR. (e) ICP-dependent determination of AUR content in liposomes. Data are presented as mean values ± SEM (n = 3 experimental replicates for panels a-e). Source data are provided as a Source Data file.

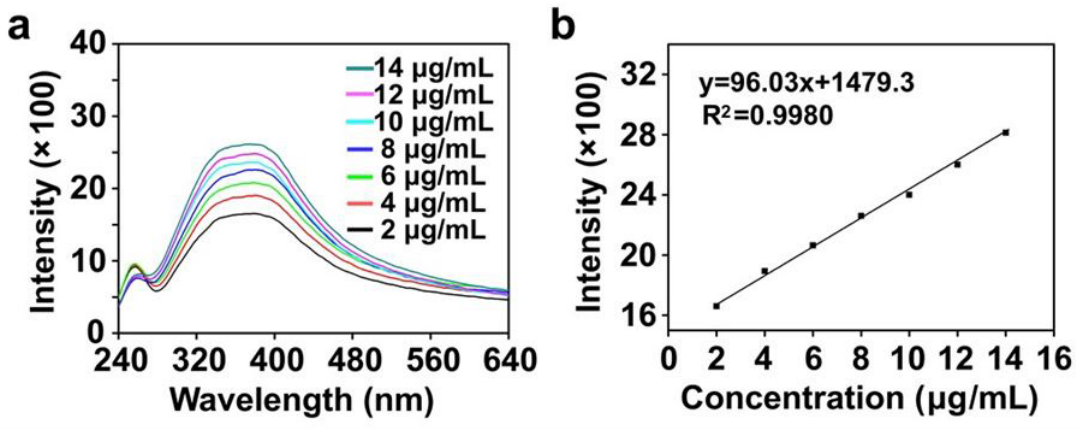

Supplementary Figure 3. Fluorescence analysis of AUR loading into the liposomes. (a) The fluorescence values of AUR at different concentrations and (b) the corresponding standard curve (n = 3 experimental replicates). Source data are provided as a Source Data file.

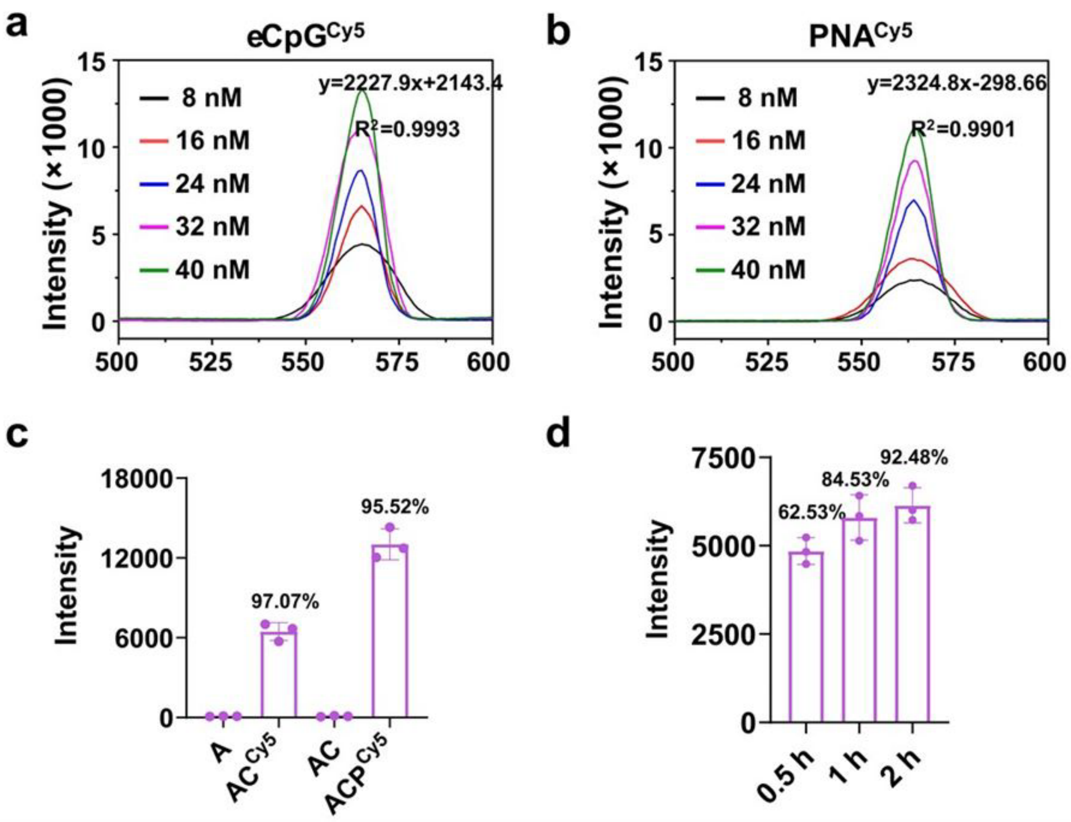

Supplementary Figure 4. Evaluation of aptamer assembly and eCpG release. (a-b) Standard curve of eCpG^{Cy5} and PNA^{Cy5} according to fluorescence analysis (n = 3 experimental replicates). (c) Assembly efficiency of AC^{Cy5} and ACP^{Cy5}. (d) Release efficiency of eCpG^{Cy5} from Lip@AUR-AC^{Cy5}P-aptPD-L1 in a time-dependent manner. Data are presented as mean values ± SEM (n = 3 experimental replicates for panels c-d). Source data are provided as a Source Data file.

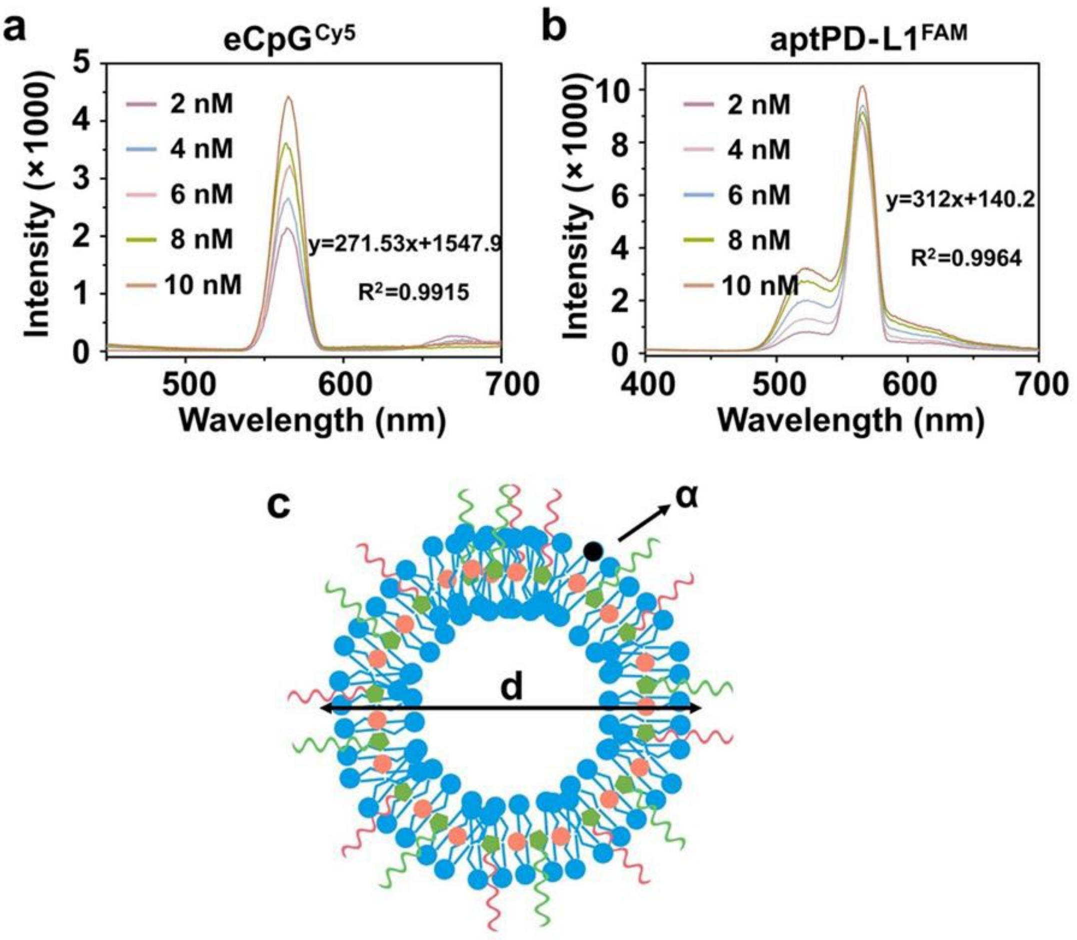

Supplementary Figure 5. Integration efficiency of ACP and aptPD-L1 in Lip@AUR-ACP-aptPD-L1. (a-b) Fluorescence spectra of $eCpG^{Cy5}$ and aptPD-$L1^{FAM}$ under different concentrations (n = 3 experimental replicates). (c) Schematic demonstration for the calculation of average number of aptamers in the liposomes. Notable parameters are described below: DNA loading = N(DNA)/N(liposomes); N(total) = $8\pi*(d/2)^2/\alpha$, N(liposomes) = C(lipid)*N_A*V/N(total). N(total) is the amount of lipids per liposome; N_A is the number of Avogadro; d is the particle diameter; α is the footprint of the lipid head group and is 0.71 nm^2 for DMPC. Source data are provided as a Source Data file.

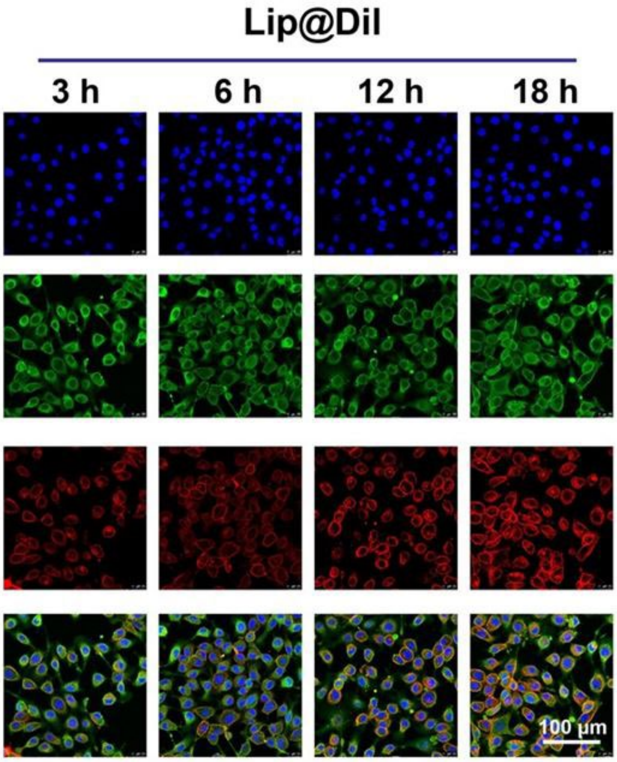

Supplementary Figure 6. Fluorescence analysis on the membrane fusion of the fusogenic liposomes. Evaluation on the membrane fusion performance of Lip@DiI with B16F10 cells and spleen cells in the co-culture system (n = 3 experimental replicates).

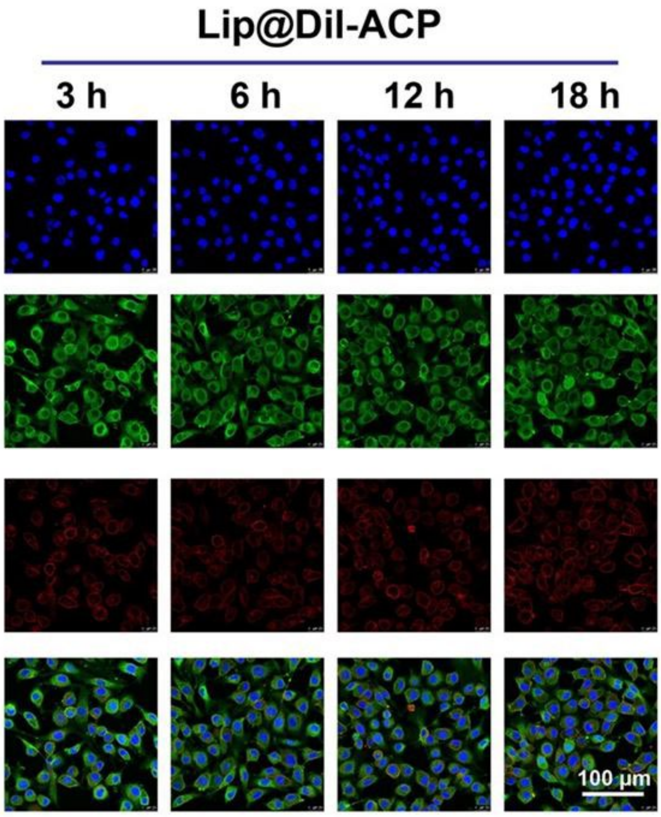

Supplementary Figure 7. Fluorescence analysis on the membrane fusion of the ACP-integrated liposomes. Membrane fusion of Lip@DiI-ACP in the co-culture system with B16F10 cells and spleen cells (n = 3 experimental replicates).

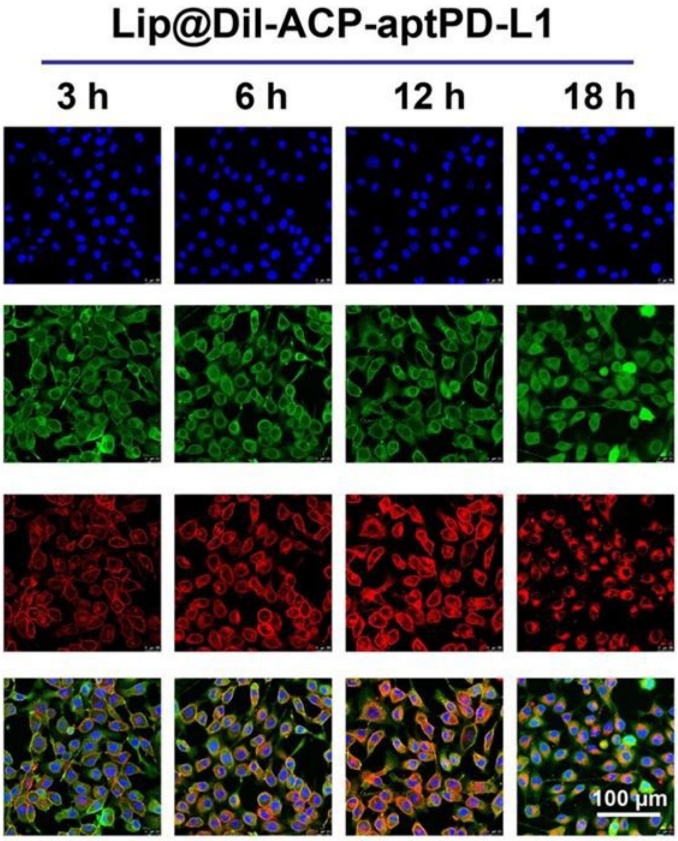

Supplementary Figure 8. Fluorescence analysis on the membrane fusion of the ACP-integrated melanoma-targeted liposomes. Membrane fusion of Lip@Dil-ACP-aptPD-L1 in the co-culture system with B16F10 cells and spleen cells (n = 3 experimental replicates).

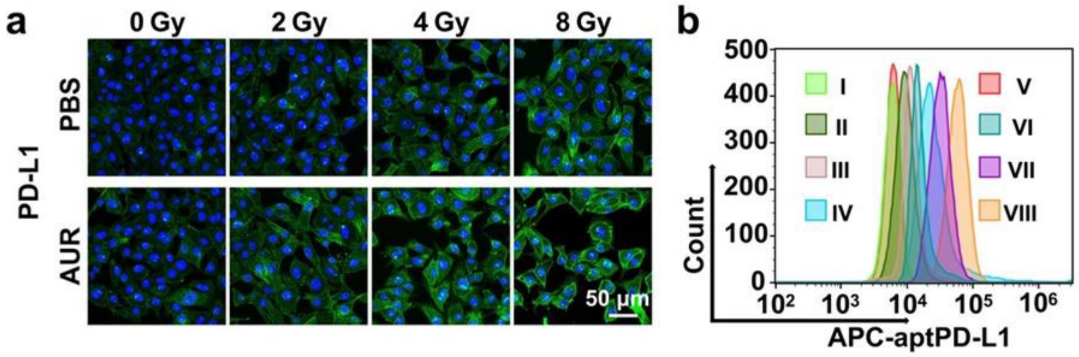

Supplementary Figure 9. Dose-dependent impact of IR on B16F10-intrinsic PD-L1 expression. (a) Confocal microscopic analysis of PD-L1 expression in AUR-treated B16F10 cells with graded IR doses (n = 3 experimental replicates). (b) Flow cytometry analysis on the expression status of PD-L1 in B16F10 cells after different treatment (n = 3 experimental replicates). I: PBS+0 Gy, II: PBS+2 Gy, III: PBS+4 Gy, IV: PBS+8 Gy, V: AUR+0 Gy, VI: AUR+2 Gy, VII: AUR+4 Gy, VIII: AUR+8 Gy.

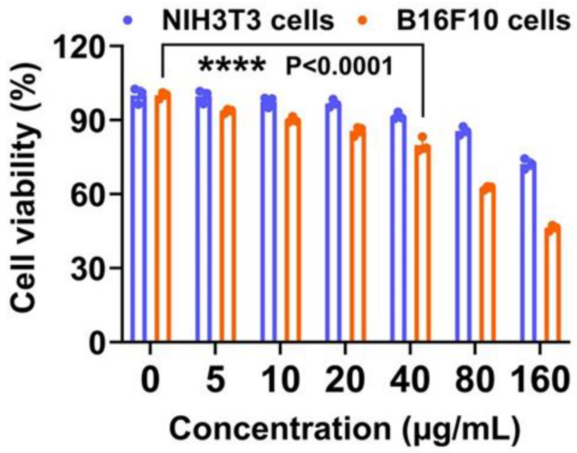

Supplementary Figure 10. Cytotoxicity profiles of the liposome systems. MTT assay regarding the cytotoxicity of Lip@AUR-aptPD-L1 on NIH3T3 cells or B16F10 cells under different concentrations. Data are presented as mean values ± SEM (n = 3 experimental replicates). Statistical analysis was carried out via one-way ANOVA method. * indicates significance at $p < 0.05$, ** indicates significance at $p < 0.01$, *** indicates significance at $p < 0.001$, **** indicates significance at $p < 0.0001$. Source data are provided as a Source Data file.

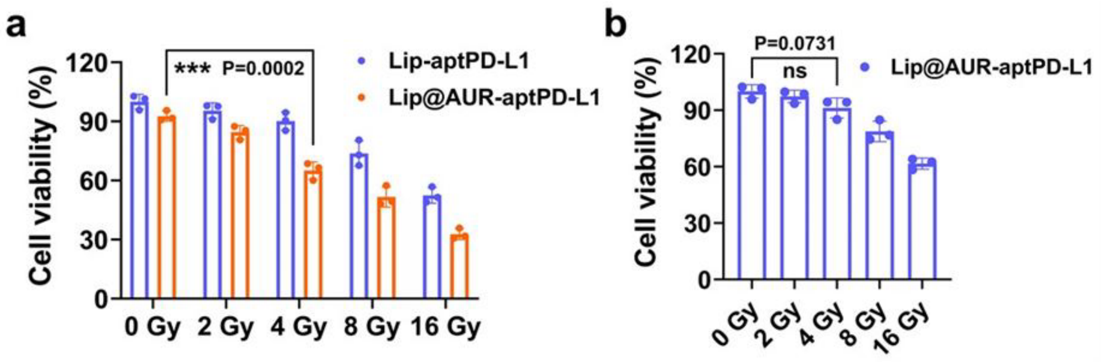

Supplementary Figure 11. Evaluation on the radiosensitizing effect of the liposomes. Impact of the combined treatment of Lip@AUR-aptPD-L1 and IR of different doses on B16F10 cells (a) and splenocytes (b). Data are presented as mean values ± SEM (n = 3 experimental replicates for panels a-b). Statistical analysis in panels a-b was carried out via one-way ANOVA method. * indicates significance at $p < 0.05$, ** indicates significance at $p < 0.01$, *** indicates significance at $p < 0.001$, **** indicates significance at $p < 0.0001$. Source data are provided as a Source Data file.

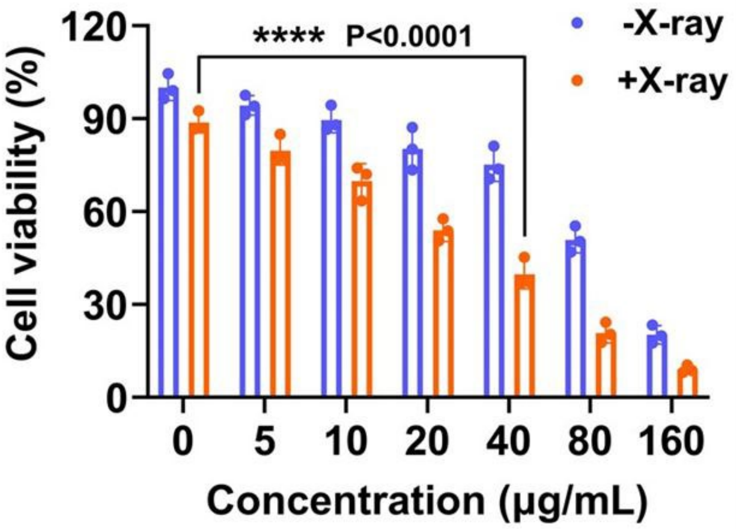

Supplementary Figure 12. Evaluation on the radio-immunotherapeutic effect of the liposomes. Cytotoxic impact of Lip@AUR-aptPD-L1 + 4 Gy IR on B16F10 cells and splenocytes in the co-culture system by MTT assay. Data are presented as mean values ± SEM (n = 3 experimental replicates). Statistical analysis carried out via one-way ANOVA method. * indicates significance at $p < 0.05$, ** indicates significance at $p < 0.01$, *** indicates significance at $p < 0.001$, **** indicates significance at $p < 0.0001$. Source data are provided as a Source Data file.

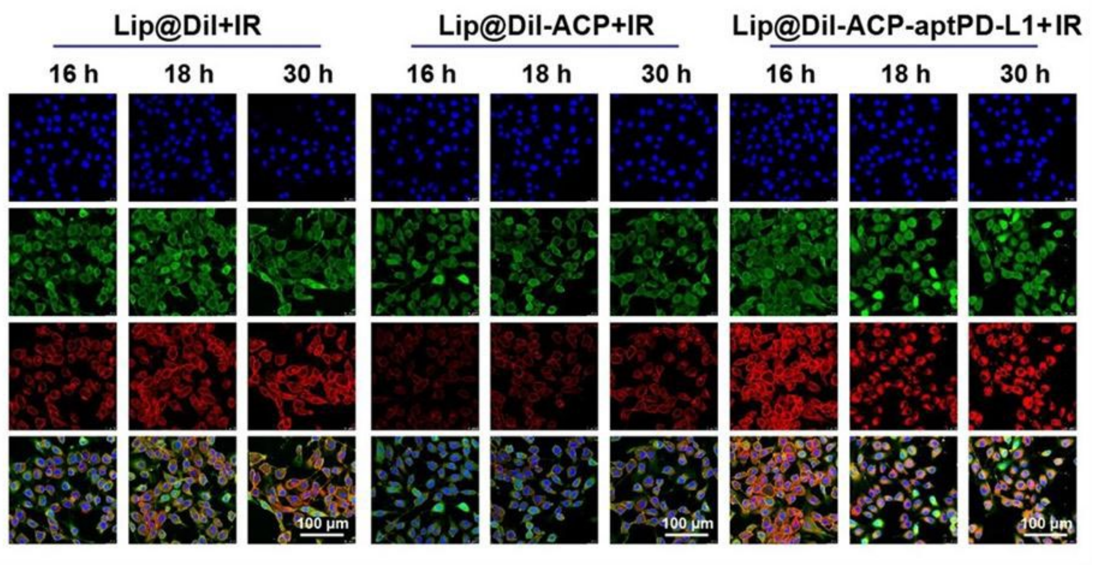

Supplementary Figure 13. Time-dependent analysis of the membrane fusion of liposomes. Comparative analysis of the membrane fusion capacity of different liposomes with 4 Gy IR to B16F10 cells under co-culture condition (n = 3 experimental replicates).

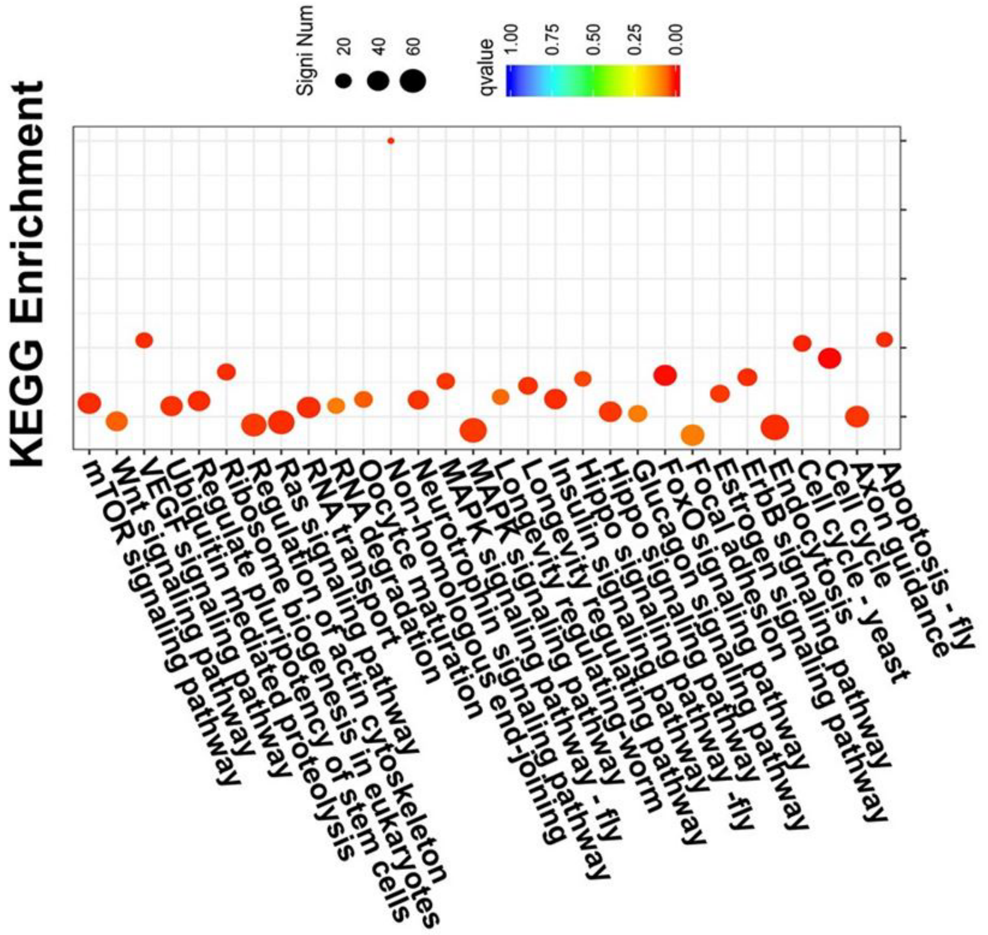

Supplementary Figure 14. Bioinformatic analysis of the treatment-induced therapeutic impact. Transcriptome sequencing analysis regarding the impact of combined Lip@AUR-aptPD-L1+ 4 Gy IR treatment on the VEGF pathway in B16F10 cells.

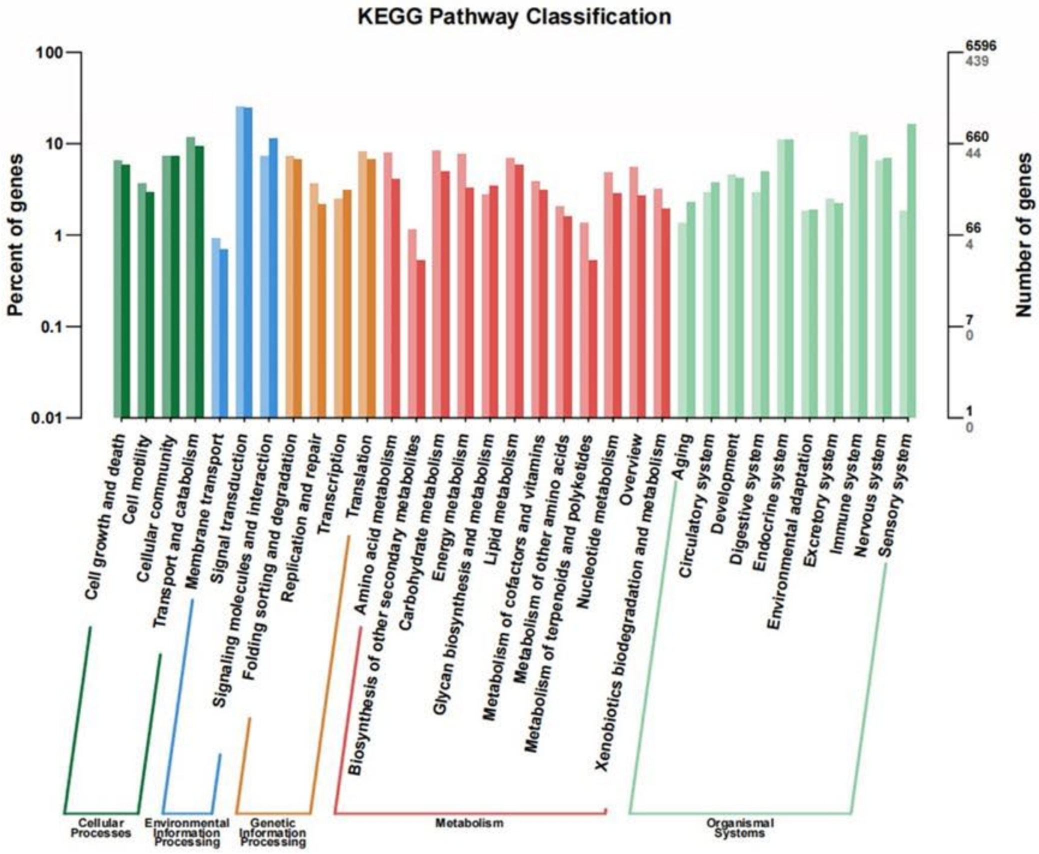
KEGG Pathway Classification
Percent of genes
Number of genes
100
10
1
0.1
0.01
6596
439
660
44
66
4
7
0
1
0
Cell growth and death
Cell motility
Cellular community
Transport and catabolism
Membrane transport
Signal transduction
Signaling molecules and interaction
Folding sorting and degradation
Replication and repair
Transcription
Translation
Amino acid metabolism
Biosynthesis of other secondary metabolites
Carbohydrate metabolism
Energy metabolism
Glycan biosynthesis and metabolism
Lipid metabolism
Metabolism of cofactors and vitamins
Metabolism of other amino acids
Metabolism of terpenoids and polyketides
Nucleotide metabolism
Overview
Xenobiotics biodegradation and metabolism
Aging
Circulatory system
Development
Digestive system
Endocrine system
Environmental adaptation
Excretory system
Immune system
Nervous system
Sensory system
Cellular Processes
Environmental Information Processing
Genetic Information Processing
Metabolism
Organismal Systems

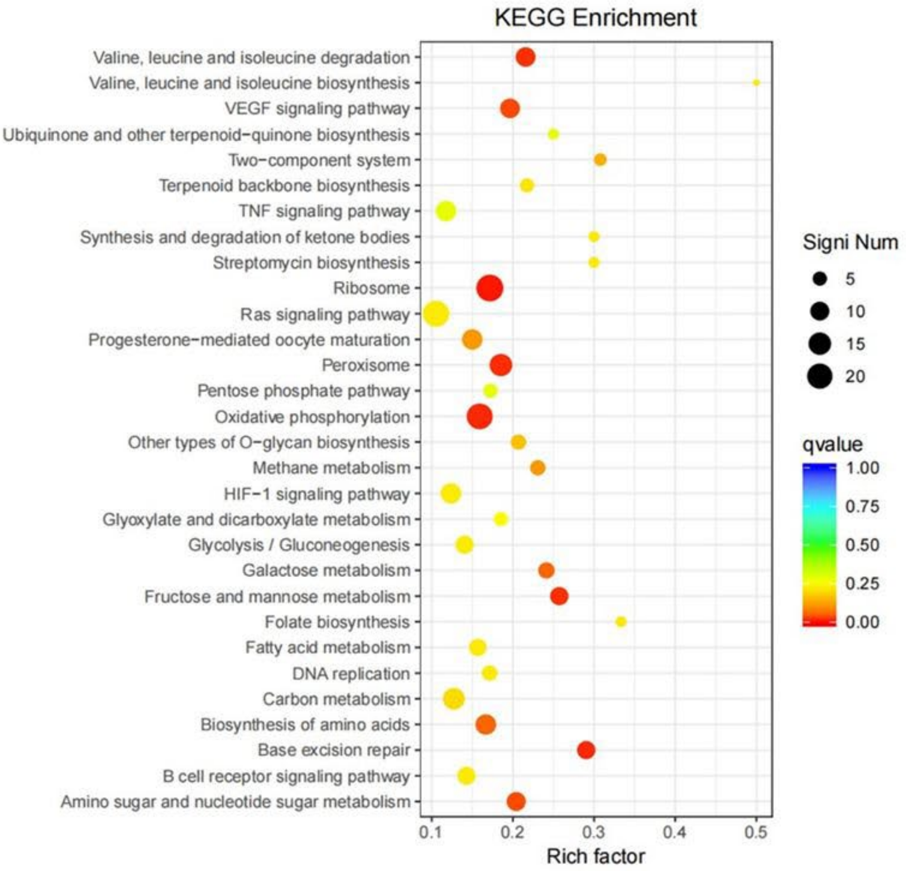
KEGG Enrichment
Valine, leucine and isoleucine degradation
Valine, leucine and isoleucine biosynthesis
VEGF signaling pathway
Ubiquinone and other terpenoid−quinone biosynthesis
Two−component system
Terpenoid backbone biosynthesis
TNF signaling pathway
Synthesis and degradation of ketone bodies
Streptomycin biosynthesis
Ribosome
Ras signaling pathway
Progesterone−mediated oocyte maturation
Peroxisome
Pentose phosphate pathway
Oxidative phosphorylation
Other types of O−glycan biosynthesis
Methane metabolism
HIF−1 signaling pathway
Glyoxylate and dicarboxylate metabolism
Glycolysis / Gluconeogenesis
Galactose metabolism
Fructose and mannose metabolism
Folate biosynthesis
Fatty acid metabolism
DNA replication
Carbon metabolism
Biosynthesis of amino acids
Base excision repair
B cell receptor signaling pathway
Amino sugar and nucleotide sugar metabolism
0.1
0.2
0.3
0.4
0.5
Rich factor
Signi Num
5
10
15
20
qvalue
1.00
0.75
0.50
0.25
0.00

Supplementary Figure 15. Transcriptome sequencing of Lip@AUR-aptPD-L1+4 Gy IR treated B16F10 cells. The down-KEGG pathway classification results and the down-KEGG enrichment results of B16F10 cells.

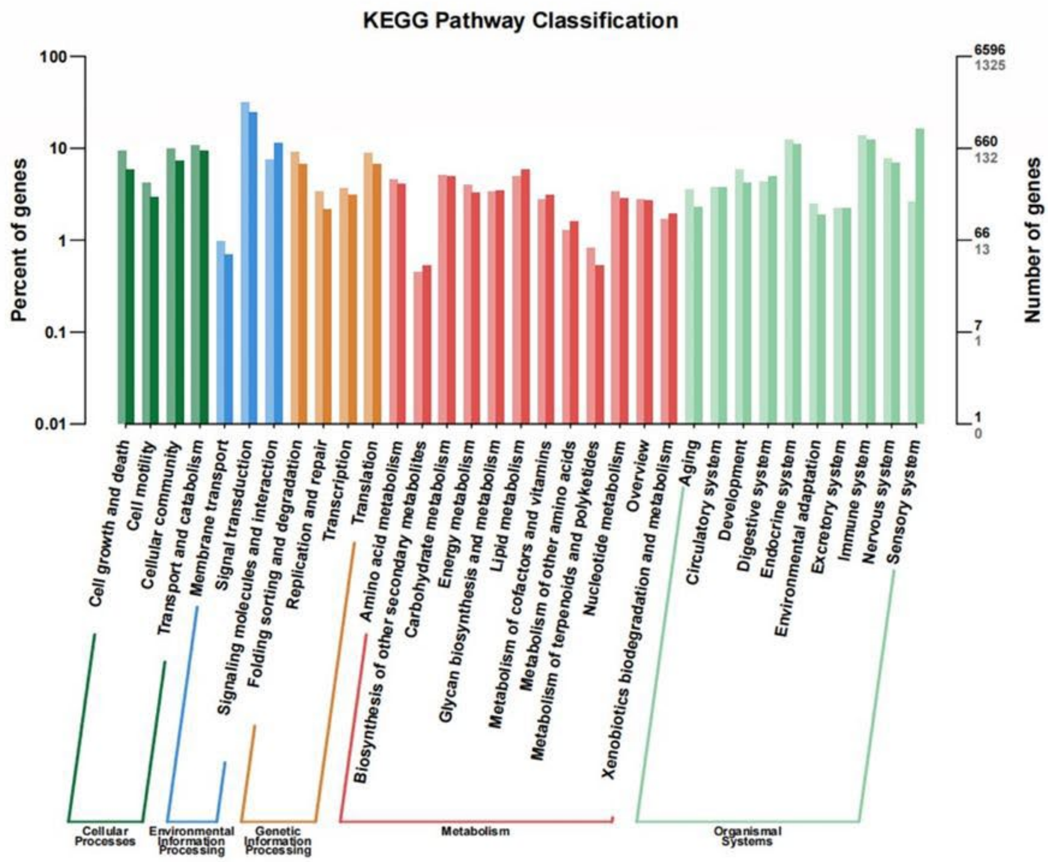
KEGG Pathway Classification
Percent of genes
Number of genes
Cell growth and death
Cell motility
Cellular community
Transport and catabolism
Membrane transport
Signal transduction
Signaling molecules and interaction
Folding sorting and degradation
Replication and repair
Transcription
Translation
Amino acid metabolism
Biosynthesis of other secondary metabolites
Carbohydrate metabolism
Energy metabolism
Glycan biosynthesis and metabolism
Lipid metabolism
Metabolism of cofactors and vitamins
Metabolism of other amino acids
Metabolism of terpenoids and polyketides
Nucleotide metabolism
Overview
Xenobiotics biodegradation and metabolism
Aging
Circulatory system
Development
Digestive system
Endocrine system
Environmental adaptation
Excretory system
Immune system
Nervous system
Sensory system
Cellular Processes
Environmental Information Processing
Genetic Information Processing
Metabolism
Organismal Systems

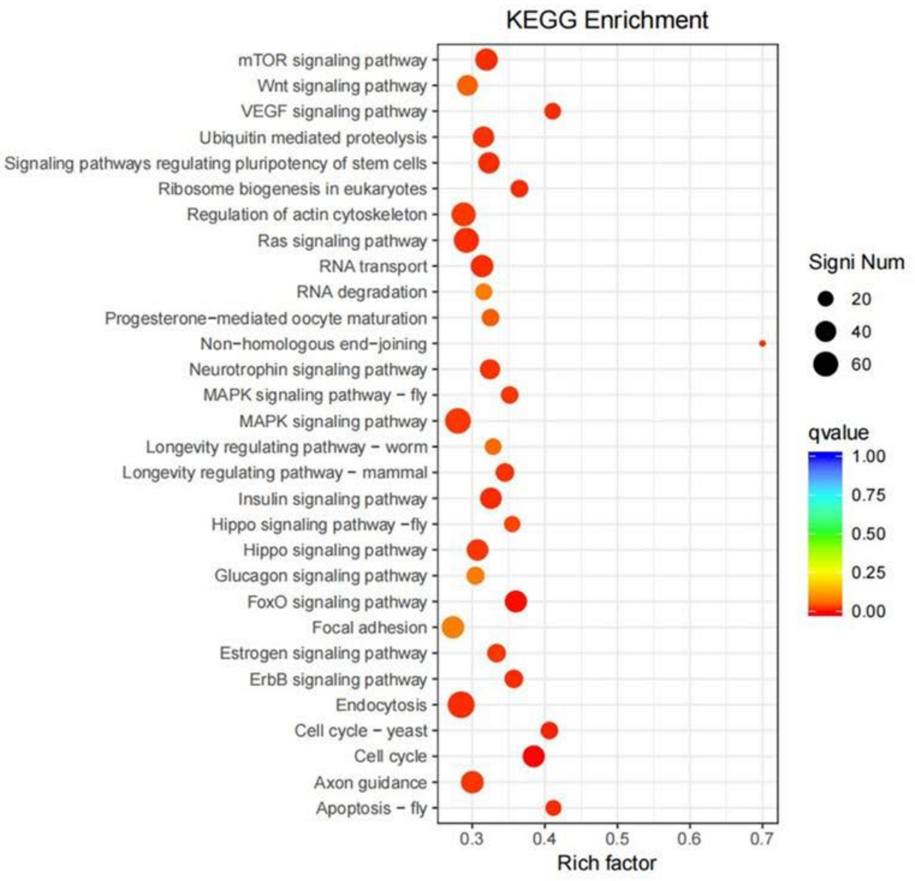
KEGG Enrichment
mTOR signaling pathway
Wnt signaling pathway
VEGF signaling pathway
Ubiquitin mediated proteolysis
Signaling pathways regulating pluripotency of stem cells
Ribosome biogenesis in eukaryotes
Regulation of actin cytoskeleton
Ras signaling pathway
RNA transport
RNA degradation
Progesterone−mediated oocyte maturation
Non−homologous end−joining
Neurotrophin signaling pathway
MAPK signaling pathway − fly
MAPK signaling pathway
Longevity regulating pathway − worm
Longevity regulating pathway − mammal
Insulin signaling pathway
Hippo signaling pathway −fly
Hippo signaling pathway
Glucagon signaling pathway
FoxO signaling pathway
Focal adhesion
Estrogen signaling pathway
ErbB signaling pathway
Endocytosis
Cell cycle − yeast
Cell cycle
Axon guidance
Apoptosis − fly
Rich factor
Signi Num
qvalue

Supplementary Figure 16. Transcriptome sequencing of Lip@AUR-aptPD-L1+4 Gy IR treated B16F10 cells. The sign-KEGG pathway classification results and the sign-KEGG enrichment results of B16F10 cells.

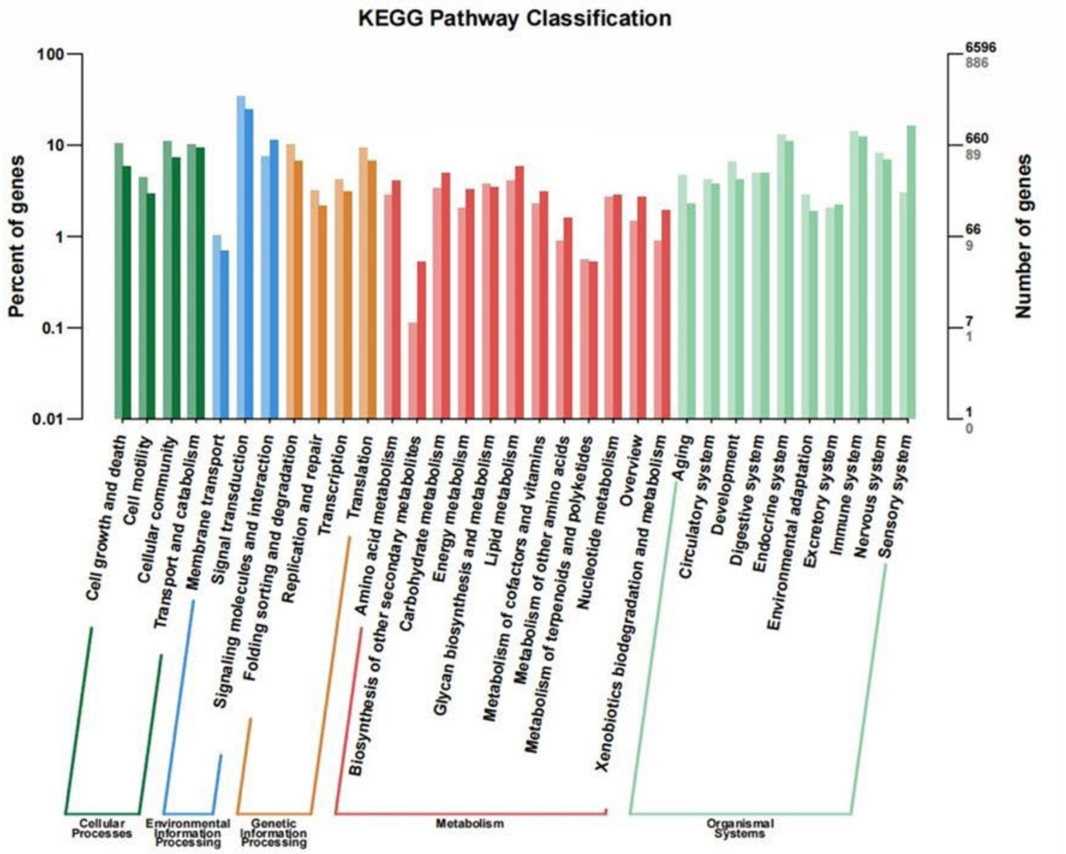
KEGG Pathway Classification
Percent of genes
Number of genes
Cell growth and death
Cell motility
Cellular community
Transport and catabolism
Membrane transport
Signal transduction
Signaling molecules and interaction
Folding sorting and degradation
Replication and repair
Transcription
Translation
Amino acid metabolism
Biosynthesis of other secondary metabolites
Carbohydrate metabolism
Energy metabolism
Glycan biosynthesis and metabolism
Lipid metabolism
Metabolism of cofactors and vitamins
Metabolism of other amino acids
Metabolism of terpenoids and polyketides
Nucleotide metabolism
Overview
Xenobiotics biodegradation and metabolism
Aging
Circulatory system
Development
Digestive system
Endocrine system
Environmental adaptation
Excretory system
Immune system
Nervous system
Sensory system
Cellular Processes
Environmental Information Processing
Genetic Information Processing
Metabolism
Organismal Systems

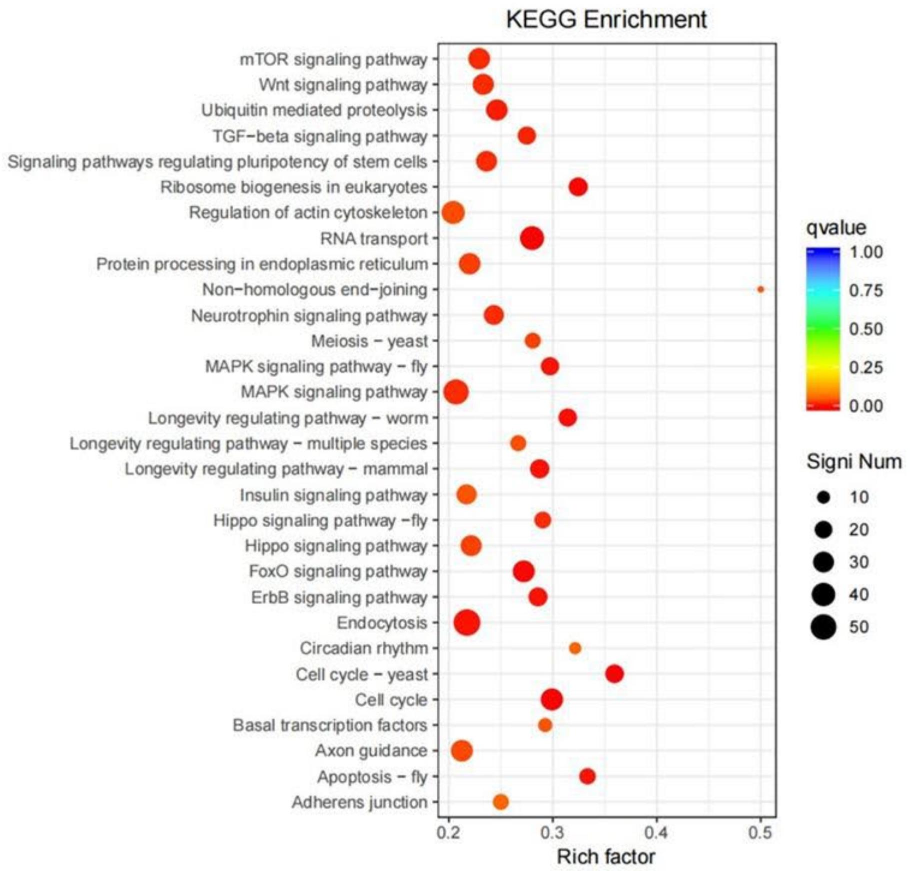
KEGG Enrichment
mTOR signaling pathway
Wnt signaling pathway
Ubiquitin mediated proteolysis
TGF−beta signaling pathway
Signaling pathways regulating pluripotency of stem cells
Ribosome biogenesis in eukaryotes
Regulation of actin cytoskeleton
RNA transport
Protein processing in endoplasmic reticulum
Non−homologous end−joining
Neurotrophin signaling pathway
Meiosis − yeast
MAPK signaling pathway − fly
MAPK signaling pathway
Longevity regulating pathway − worm
Longevity regulating pathway − multiple species
Longevity regulating pathway − mammal
Insulin signaling pathway
Hippo signaling pathway −fly
Hippo signaling pathway
FoxO signaling pathway
ErbB signaling pathway
Endocytosis
Circadian rhythm
Cell cycle − yeast
Cell cycle
Basal transcription factors
Axon guidance
Apoptosis − fly
Adherens junction
qvalue
Signi Num
Rich factor

Supplementary Figure 17. Transcriptome sequencing of Lip@AUR-aptPD-L1+4 Gy IR treated B16F10 cells. The up-KEGG pathway classification results and the up-KEGG enrichment results of B16F10 cells.

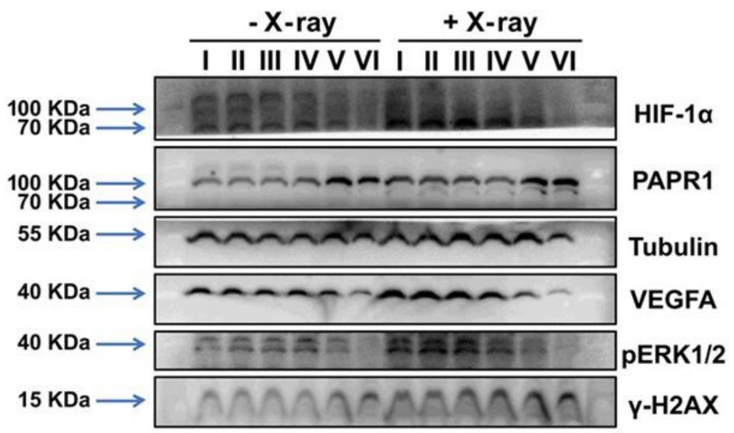

Supplementary Figure 18. Western Blot analysis on the biochemical changes in IR-treated B16F10 cells in vitro. Western Blot analysis on the expression levels of key proteins related to IR damage and ERK1/2-HIF-1α-VEGF pathway (n = 3 experimental replicates). I: PBS, II: Lip, III: Lip-aptPD-L1, IV: Lip-ACP-aptPD-L1, V: Lip@AUR-aptPD-L1, VI: Lip@AUR-ACP-aptPD-L1. Source data are provided as a Source Data file.

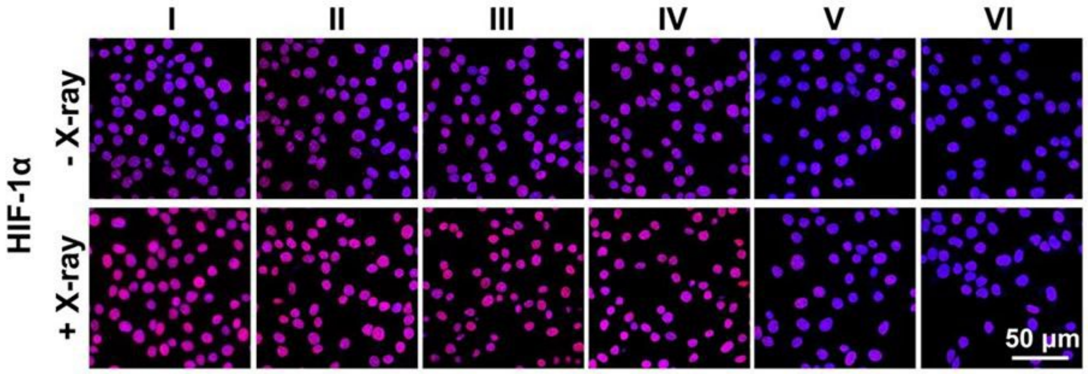

Supplementary Figure 19. Immunofluorescence analysis on AUR-mediated suppression of HIF-1α expression. Expression of HIF-1α in the B16F10 nucleus after different treatment (n = 3 experimental replicates). I: PBS, II: Lip, III: Lip-aptPD-L1, IV: Lip-ACP-aptPD-L1, V: Lip@AUR-aptPD-L1, VI: Lip@AUR-ACP-aptPD-L1.

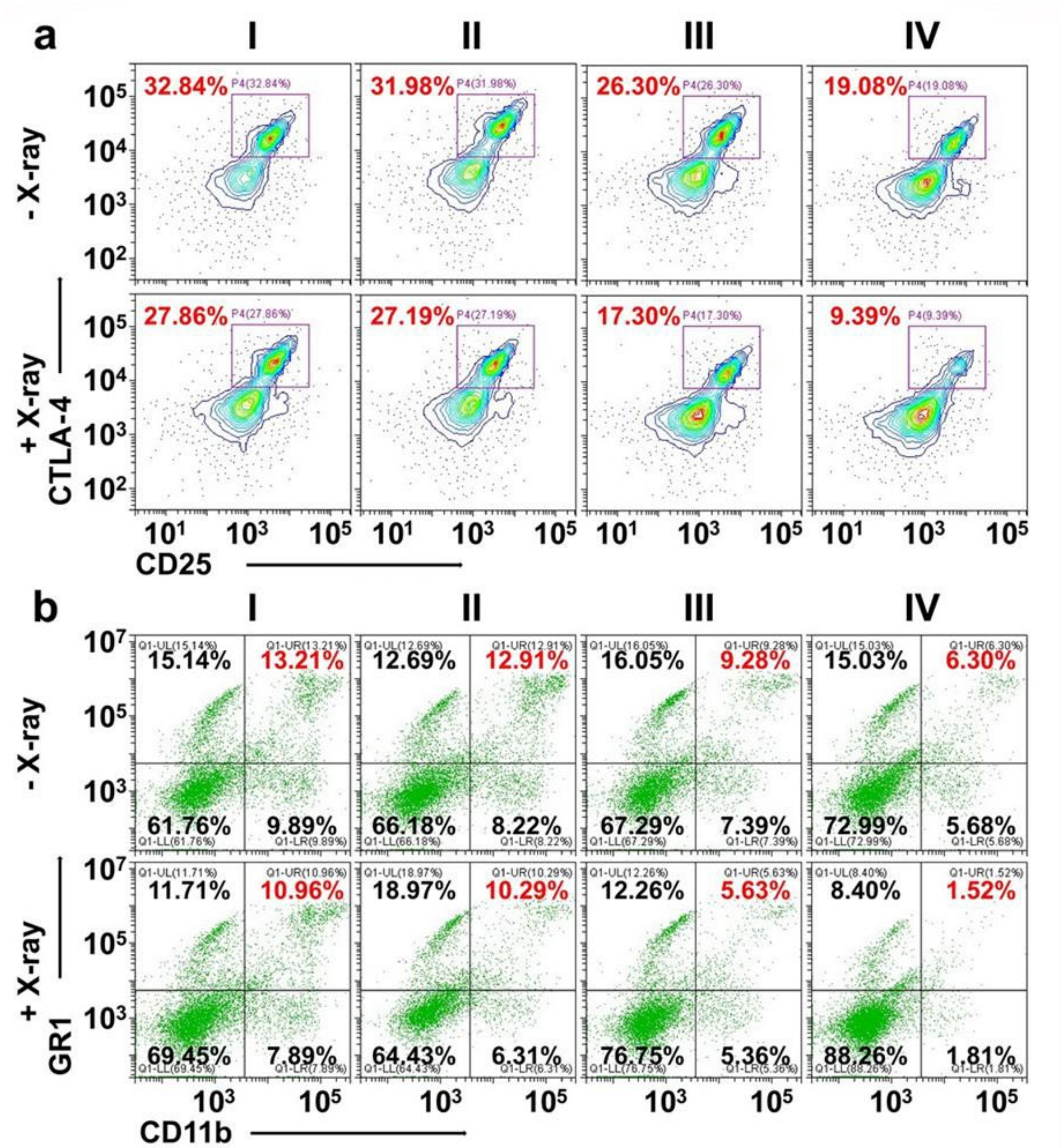

Supplementary Figure 20. Evaluation on the treatment-induced inhibition of major immunosuppressor cell populations. (a) Frequencies of Tregs (CD4+CD25+CTLA-4+) in the co-culture system after treatment with different groups (n = 3 experimental replicates). (b) Frequencies of MDSCs (CD11b+GR1+) in the co-culture system after treatment with different groups. I: PBS, II: Lip, III: Lip@AUR, IV: Lip@AUR-aptPD-L1 (n = 3 experimental replicates).

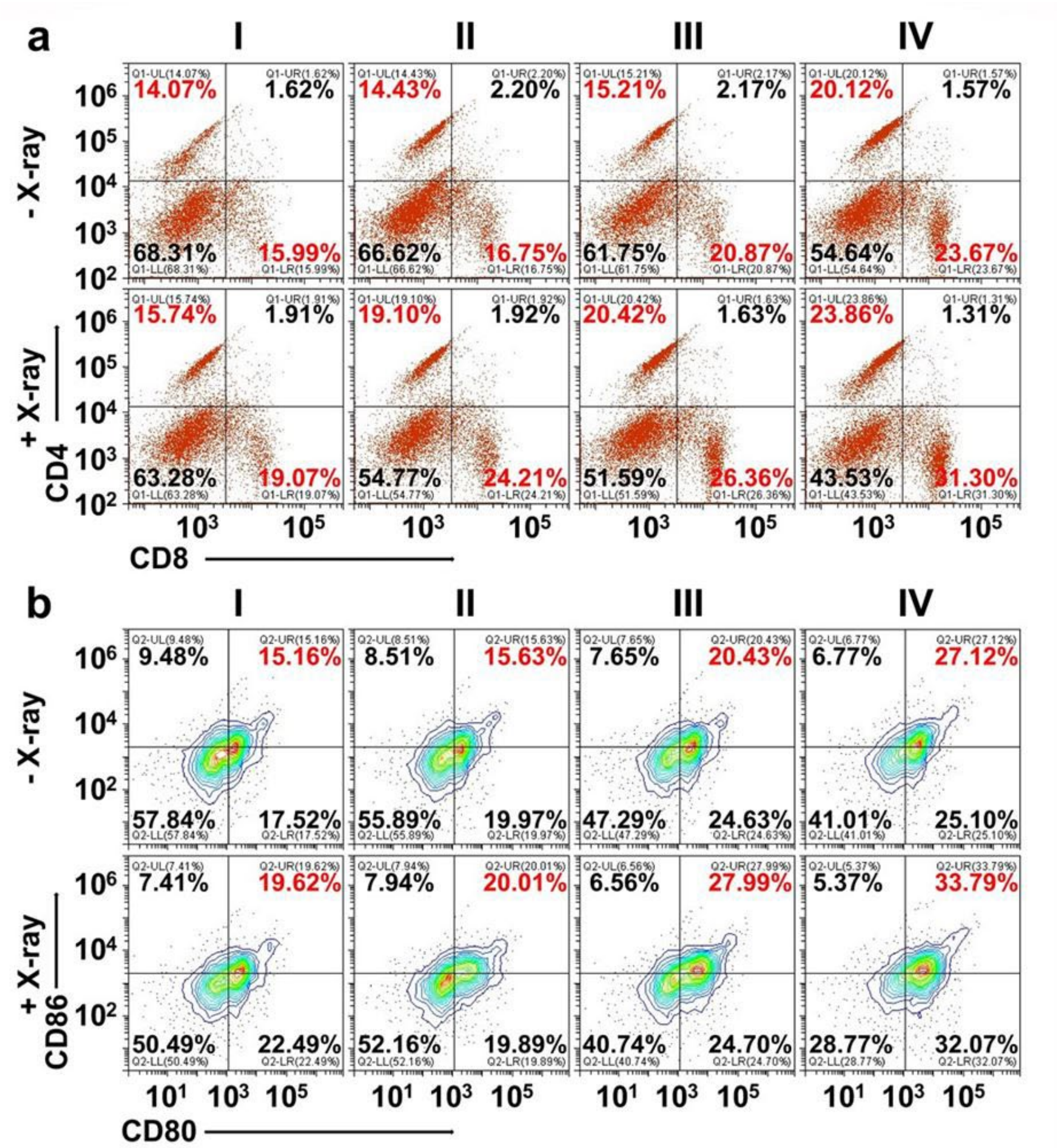

Supplementary Figure 21. Evaluation on the treatment-induced expansion of major cell populations related to the adaptive immunity. (a) Frequencies of effector T cells (CD3+CD4+CD8+) in the co-culture system after treatment with different groups (n = 3 experimental replicates). (b) Frequencies of DCs (CD11c+CD80+CD86+) in the co-culture system after treatment with different groups (n = 3 experimental replicates). I: PBS, II: Lip, III: Lip@AUR, IV: Lip@AUR-aptPD-L1.

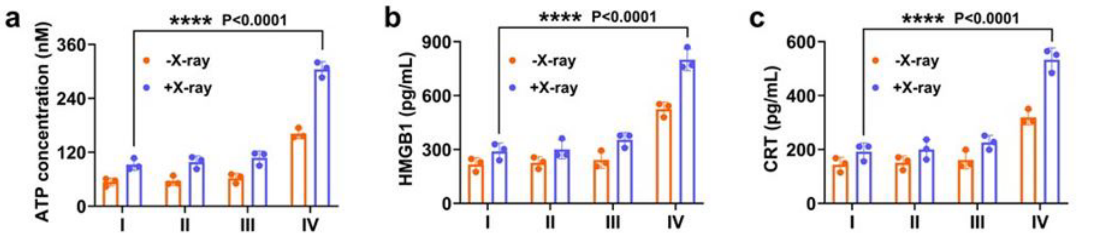

Supplementary Figure 22. Quantitative analysis of treatment-induced release of key DAMPs. The group set-ups include (a) ATP, (b) HMGB1 and (c) CRT. I: PBS, II: Lip, III: Lip-aptPD-L1, IV: Lip@AUR-aptPD-L1. Data are presented as mean values ± SEM (n = 3 experimental replicates for panels a-c). Statistical analysis in panels a-c was carried out via one-way ANOVA method. * indicates significance at $p < 0.05$, ** indicates significance at $p < 0.01$, *** indicates significance at $p < 0.001$, **** indicates significance at $p < 0.0001$. Source data are provided as a Source Data file.

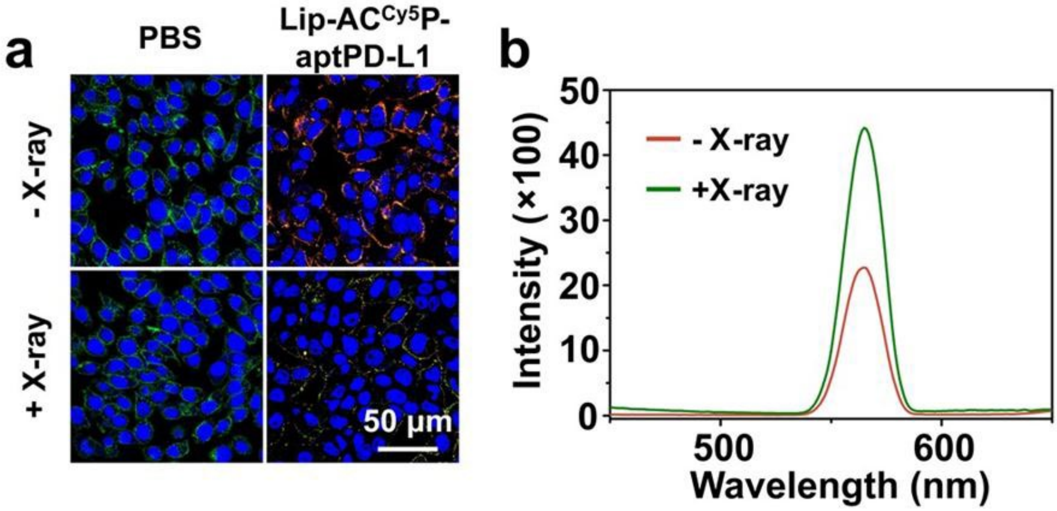

Supplementary Figure 23. AND-gate ACP activation from Lip-AC^{Cy5}P-aptPD-L1+ 4 Gy IR treated B16F10 cells. (a) The release of eCpG^{Cy5} from B16F10 cell surface by CLSM imaging (n = 3 experimental replicates). (b) The release of eCpG^{Cy5} from B16F10 cell surface by fluorescence spectroscopy (n = 3 experimental replicates). Source data are provided as a Source Data file.

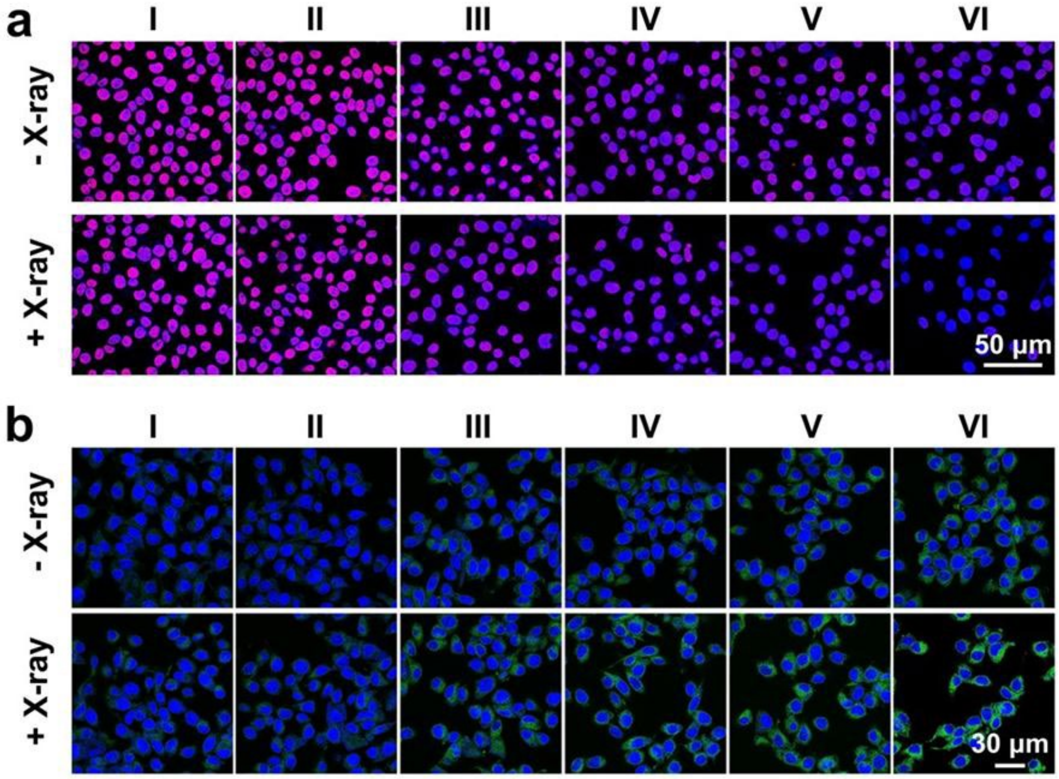

Supplementary Figure 24. Treatment-induced ICD of B16F10 cells in the co-culture system. (a) The HMGB1 expression in the B16F10 cell nucleus (red fluorescence) (n = 3 experimental replicates). (b) The CRT expression on cell membrane (green fluorescence) (n = 3 experimental replicates). I: PBS, II: Lip, III: Lip-aptPD-L1, IV: Lip-ACP-aptPD-L1, V: Lip@AUR-aptPD-L1, VI: Lip@AUR-ACP-aptPD-L1.

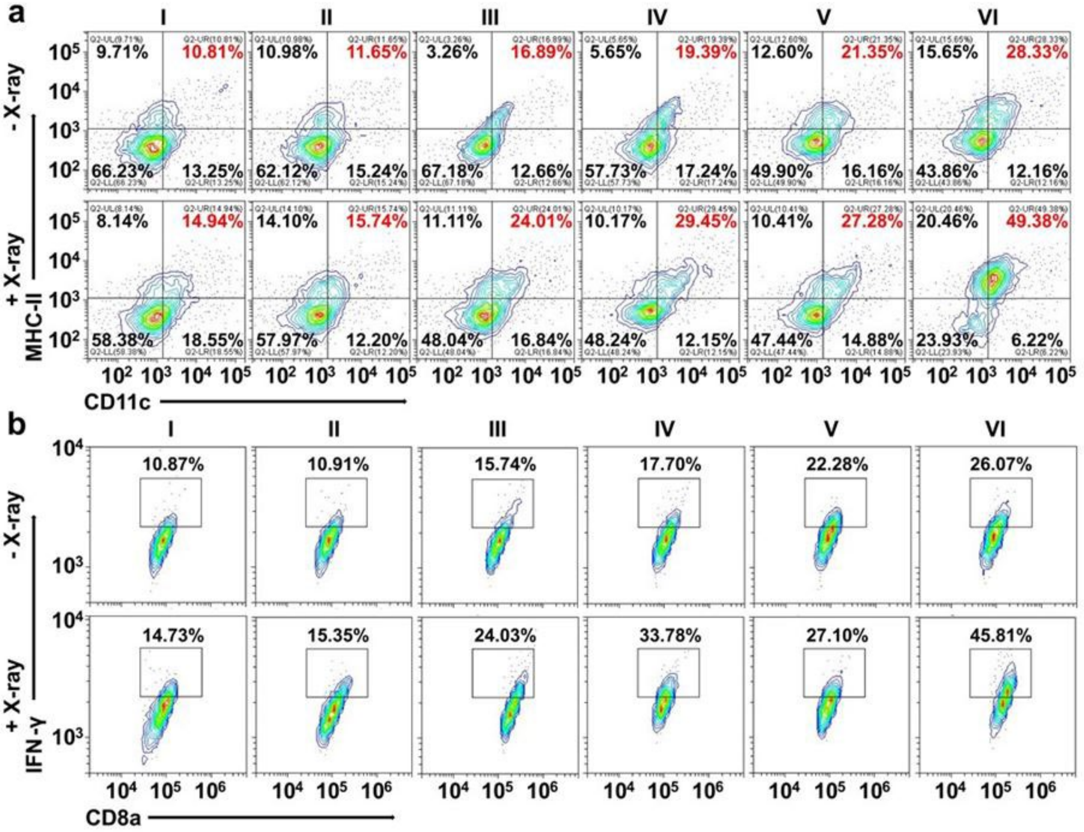

Supplementary Figure 25. Treatment-induced stimulatory effect on the adaptive immune system. (a) Frequencies of DCs (CD11c+MHC-II+) in the co-culture system after treatment with different groups (n = 3 experimental replicates). (b) Frequencies of cytotoxic CD8+T cells (CD8a+IFN-γ+) in the co-culture system after treatment with different groups (n = 3 experimental replicates). I: PBS, II: Lip, III: Lip-aptPD-L1, IV: Lip-ACP-aptPD-L1, V: Lip@AUR-aptPD-L1, VI: Lip@AUR-ACP-aptPD-L1.

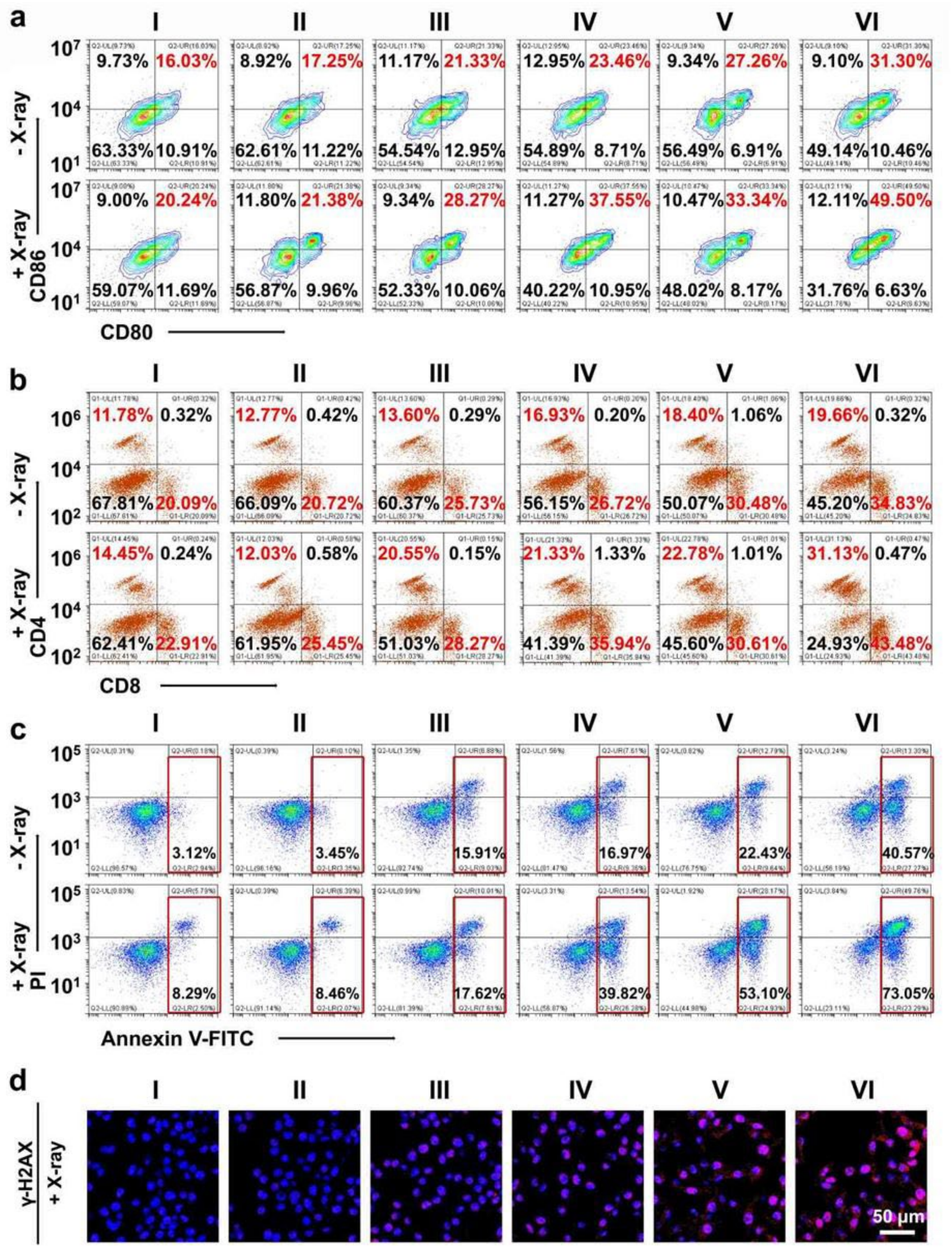

Supplementary Figure 26. The second batch of experimental replicates for desiccating the immunostimulatory effect of combined Lip@AUR-ACP-aptPD-L1 and 4 Gy IR treatment in vitro. (a) Flow cytometry analysis on the maturation status (CD11c+CD80+CD86+) of DCs in the co-incubation system after different treatments. Experimental replicate of the data shown in Figure 5a (n = 3 experimental replicates).

(b) Flow cytometry analysis on T cell activation status (CD3+CD4+CD8+) in the co-incubation system after different treatments. Experimental replicate of the data shown in Figure 5b (n = 3 experimental replicates). (c) Flow cytometry analysis on the apoptosis of B16F10 cells after different treatments in co-culture system. Experimental replicate of the data shown in Figure 5g (n = 3 experimental replicates). (d) γ-H2AX immunofluorescence of IR-treated B16F10 cells after different sample treatments. Experimental replicate of the data shown in Figure 5h. (n = 3 experimental replicates). I: PBS, II: Lip, III: Lip-aptPD-L1, IV: Lip-ACP-aptPD-L1, V: Lip@AUR-aptPD-L1, VI: Lip@AUR-ACP-aptPD-L1.

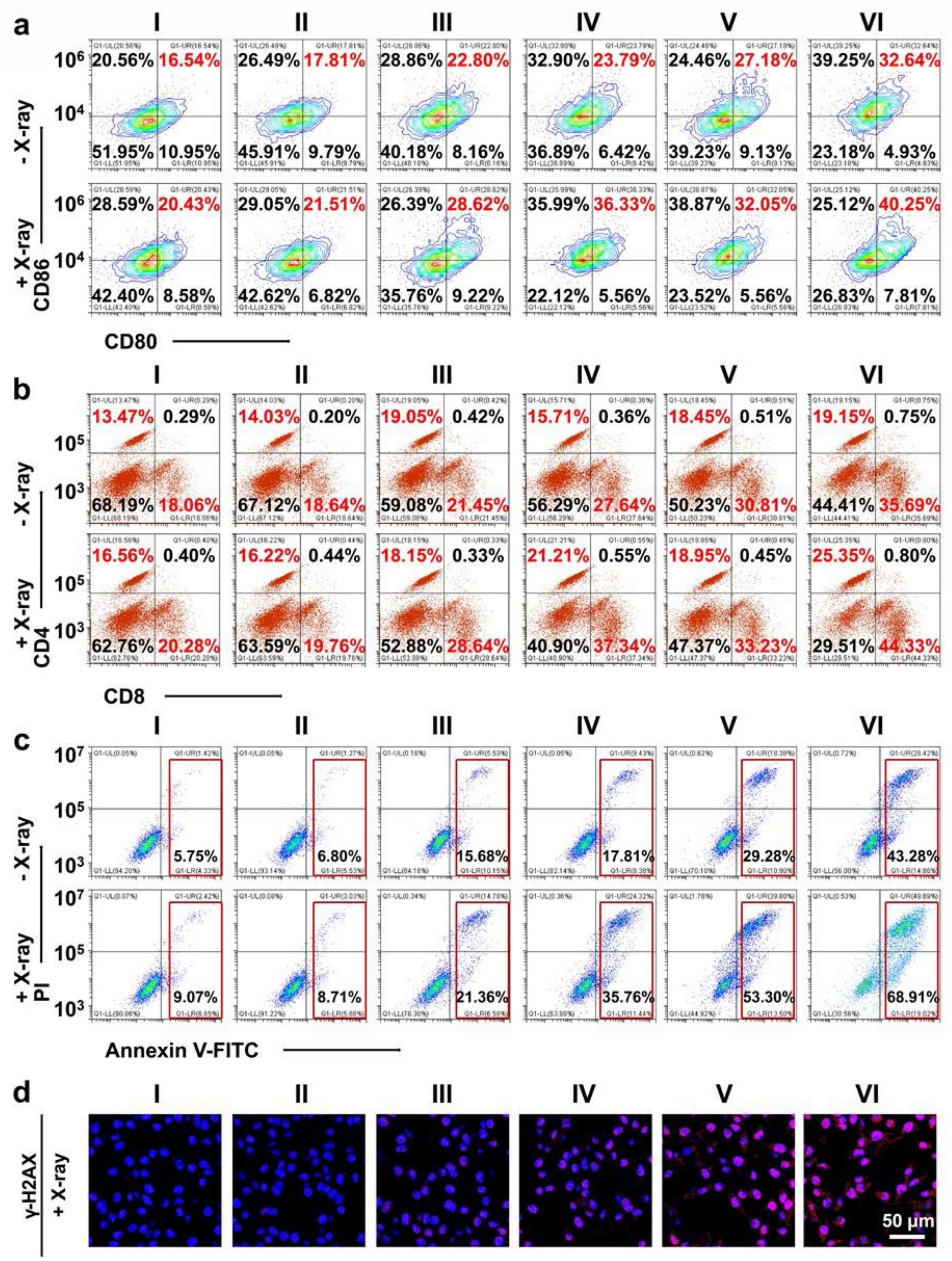

Supplementary Figure 27. The third batch of experimental replicates for desiccating the immunostimulatory effect of combined Lip@AUR-ACP-aptPD-L1 and 4 Gy IR treatment in vitro. (a) Flow cytometry analysis on the maturation status (CD11c+CD80+CD86+) of DCs in the co-incubation system after different treatments. Experimental replicate of the data shown in Figure 5a (n = 3 experimental replicates).

(b) Flow cytometry analysis on T cell activation status (CD3+CD4+CD8+) in the co-incubation system after different treatments. Experimental replicate of the data shown in Figure 5b (n = 3 experimental replicates). (c) Flow cytometry analysis on the apoptosis of B16F10 cells after different treatments in co-culture system. Experimental replicate of the data shown in Figure 5g (n = 3 experimental replicates). (d) γ-H2AX immunofluorescence of IR-treated B16F10 cells after different sample treatments. Experimental replicate of the data shown in Figure 5h. (n = 3 experimental replicates). I: PBS, II: Lip, III: Lip-aptPD-L1, IV: Lip-ACP-aptPD-L1, V: Lip@AUR-aptPD-L1, VI: Lip@AUR-ACP-aptPD-L1.

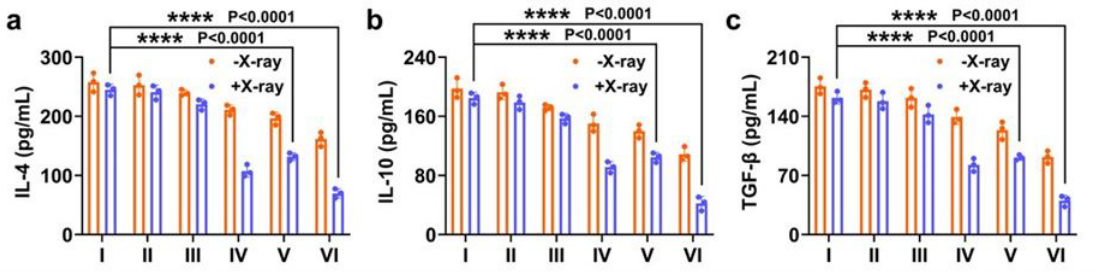

Supplementary Figure 28. Evaluation on the treatment-induced changes in the secretion of anti-inflammatory factors. The secretion levels of various anti-inflammatory factors including (a) IL-4, (b) IL-10 and (c) TGF-β after treatment with different materials in the co-culture system. I: PBS, II: Lip, III: Lip-aptPD-L1, IV: Lip-ACP-aptPD-L1, V: Lip@AUR-aptPD-L1, VI: Lip@AUR-ACP-aptPD-L1. Data are presented as mean values ± SEM (n = 3 experimental replicates for panels a-c). Statistical analysis in panels a-c was carried out via one-way ANOVA method. * indicates significance at $p < 0.05$, ** indicates significance at $p < 0.01$, *** indicates significance at $p < 0.001$, **** indicates significance at $p < 0.0001$. Source data are provided as a Source Data file.

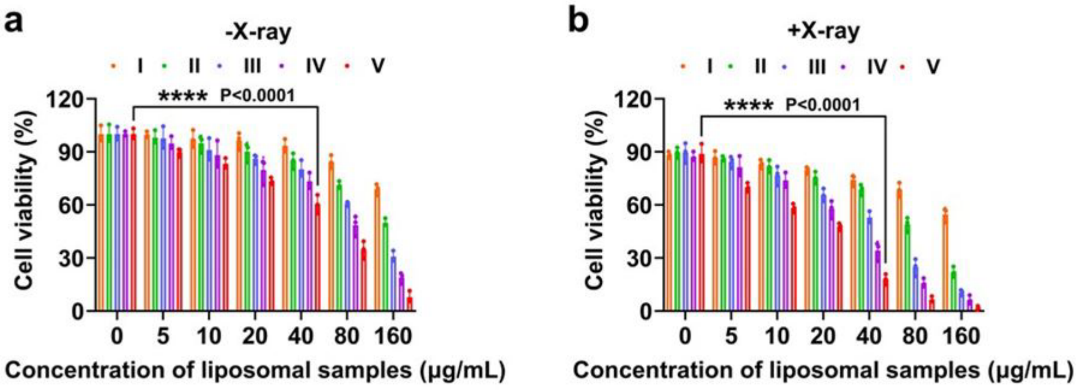

Supplementary Figure 29. MTT assay on the radio-immunotherapeutic effect of the liposomes in vitro. B16F10 inhibition effect of different groups (a) without or (b) with 4 Gy IR under the co-culture condition by MTT assay. I: Lip, II: Lip-aptPD-L1, III: Lip-ACP-aptPD-L1, IV: Lip@AUR-aptPD-L1, V: Lip@AUR-ACP-aptPD-L1. Data are presented as mean values ± SEM (n = 3 experimental replicates for panels a-b). Statistical analysis in panels a-b was carried out via one-way ANOVA method. * indicates significance at $p < 0.05$, ** indicates significance at $p < 0.01$, *** indicates significance at $p < 0.001$, **** indicates significance at $p < 0.0001$. Source data are provided as a Source Data file.

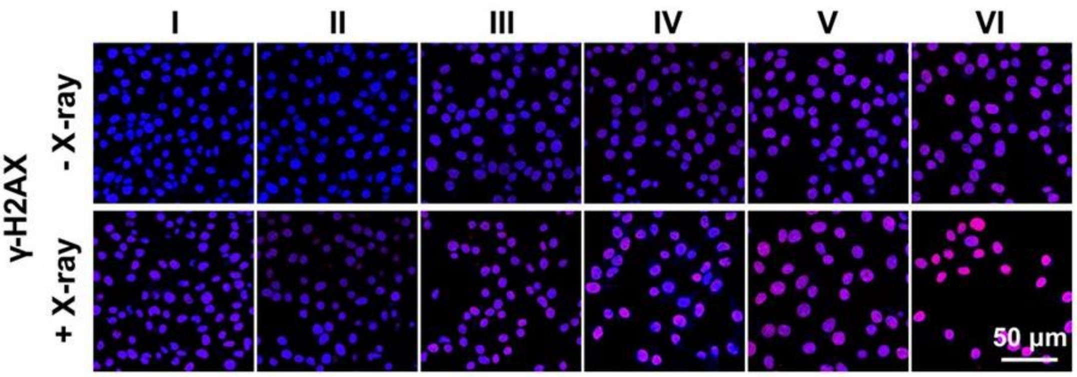

Supplementary Figure 30. Radiosensitization effect of different samples on B16F10 cells in vitro by detecting γ-H2AX immunofluorescence. The group set-ups are I: PBS, II: Lip, III: Lip-aptPD-L1, IV: Lip-ACP-aptPD-L1, V: Lip@AUR-aptPD-L1, VI: Lip@AUR-ACP-aptPD-L1 (n = 3 experimental replicates).

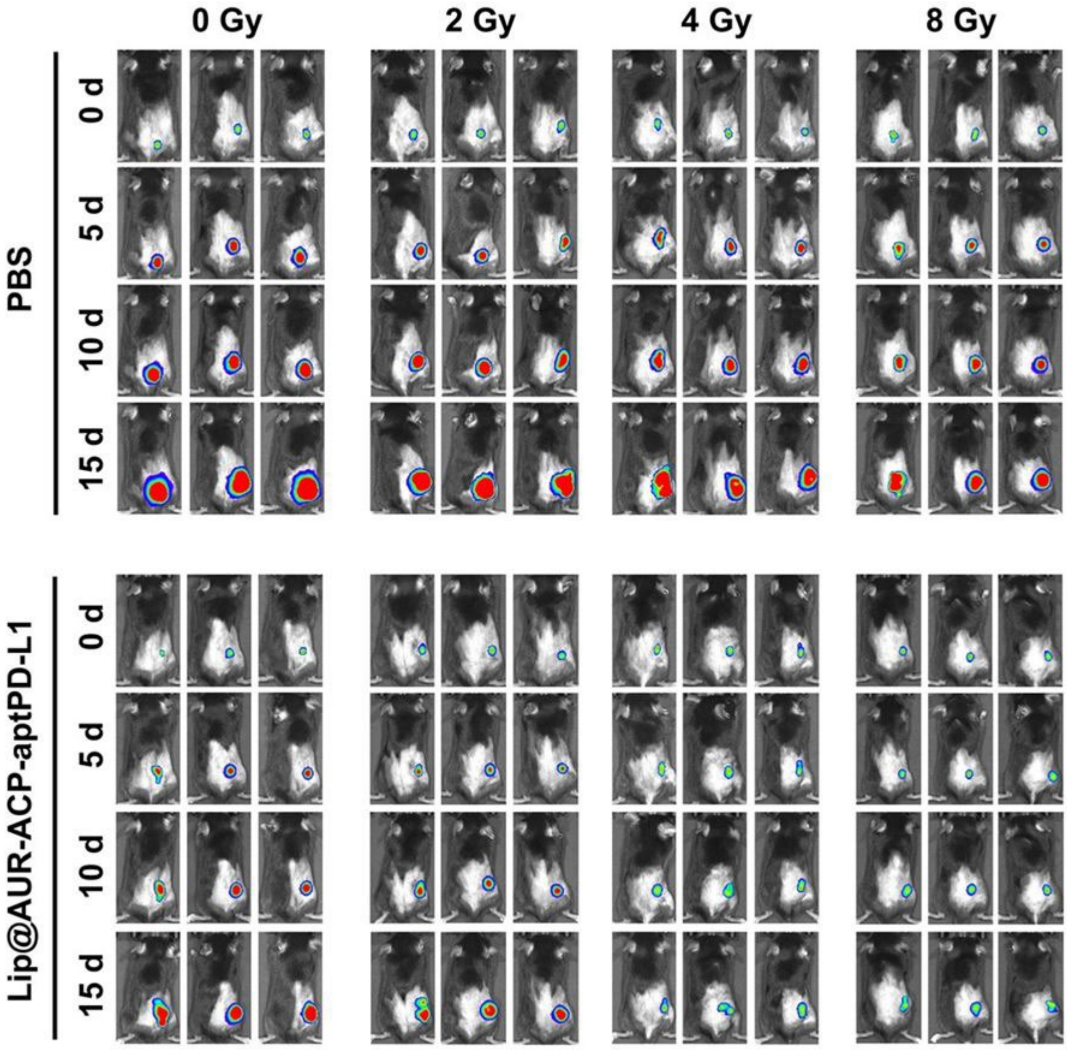

Supplementary Figure 31. Evaluation on the effects of IR doses on the therapeutic potency in vivo. In vivo bioluminescence images of B16F10-luc tumor-bearing mice with different IR doses with three mice per group.

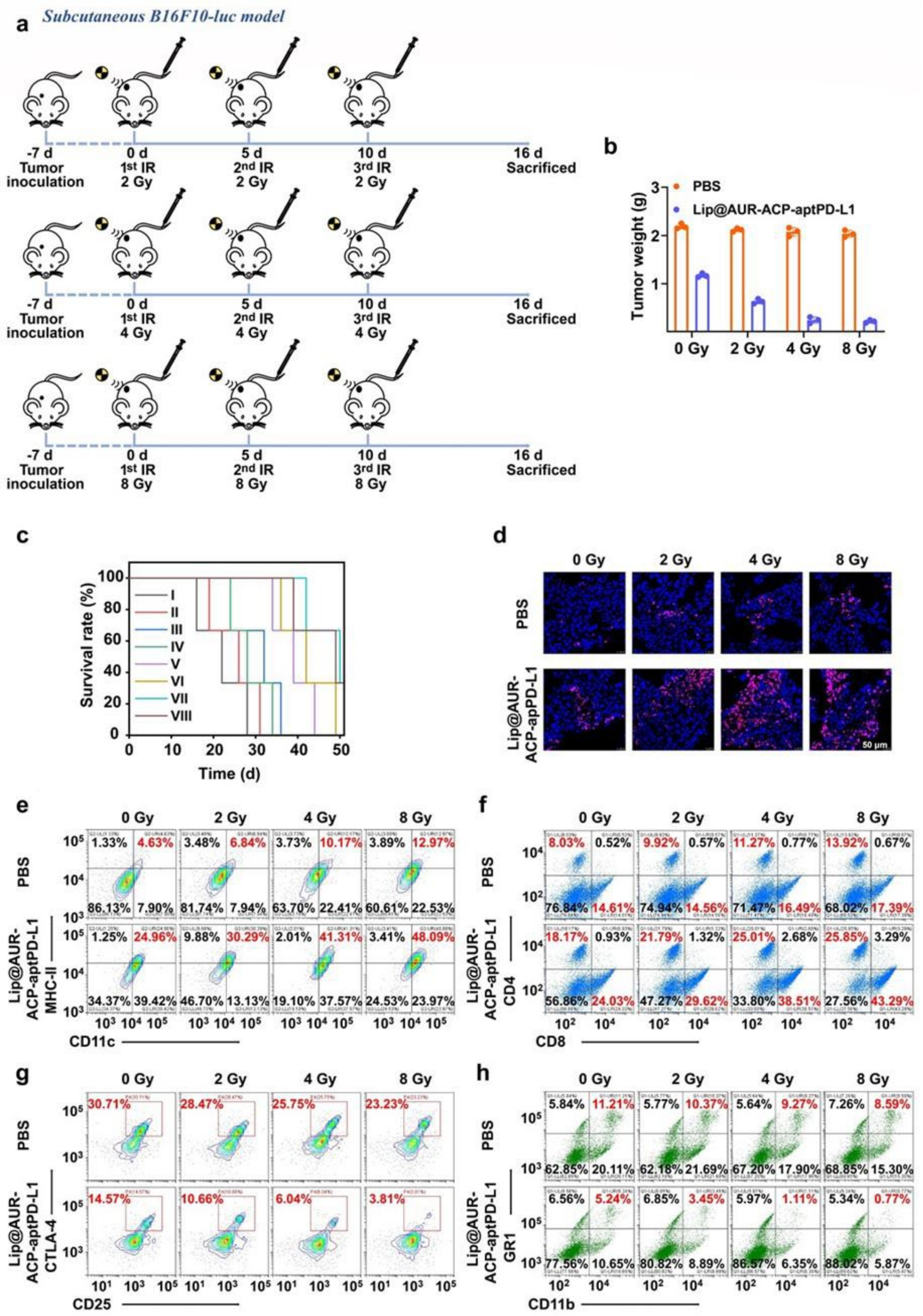

Supplementary Figure 32. Evaluation on the effects of IR doses on the therapeutic potency in vivo. (a) Schematic representation of the treatment protocol for B16F10-luc tumor-bearing mice with graded IR doses. (b) Final tumor weight analysis after the 15-

day treatment. (c) Survival curve of B16F10 tumor-bearing mice after different treatments. I: PBS+0 Gy, II: PBS+2 Gy, III: PBS+4 Gy, IV: PBS+8 Gy, V: Lip@AUR-ACP-aptPD-L1+0 Gy, VI: Lip@AUR-ACP-aptPD-L1+2 Gy, VII: Lip@AUR-ACP-aptPD-L1+4 Gy, VIII: Lip@AUR-ACP-aptPD-L1+8 Gy. (d) TUNEL staining of B16F10 tumor tissue samples after different treatments with three mice per group. (e-h) Flow cytometry analysis on the infiltration of DCs (CD11c+MHC-II+), effector T cells (CD3+CD4+CD8+), Tregs (CD4+CD25+CTLA-4+) and MDSCs (CD11b+GR1+) in the B16F10 tumors after different treatments with three mice per group. Data are presented as mean values ± SEM (n = 3 mice for panels b-c). Source data are provided as a Source Data file.

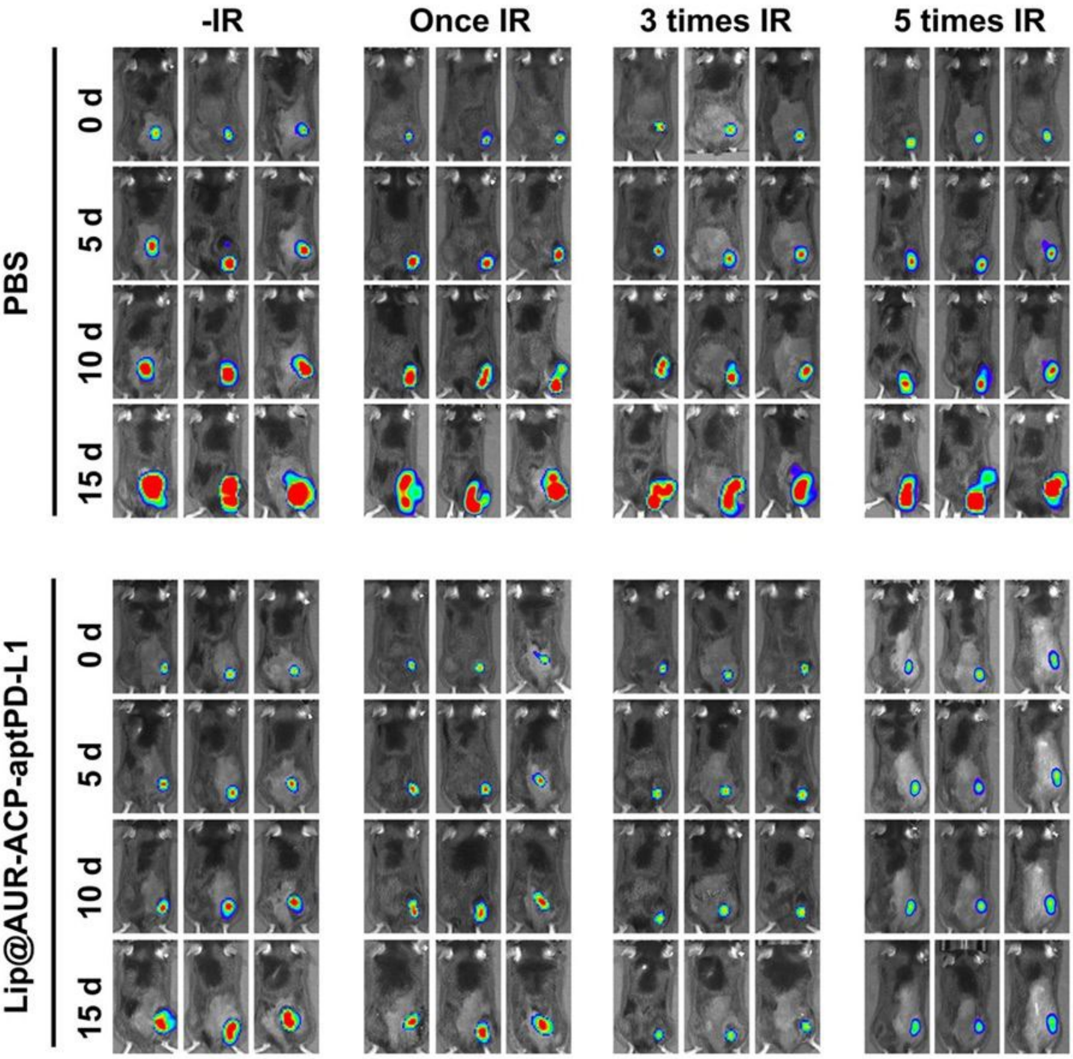

Supplementary Figure 33. Evaluation on the effect of IR fractionations on the therapeutic potency in vivo. In vivo bioluminescence images of B16F10-luc tumor-bearing mice under different fractionation set-ups with three mice per group.

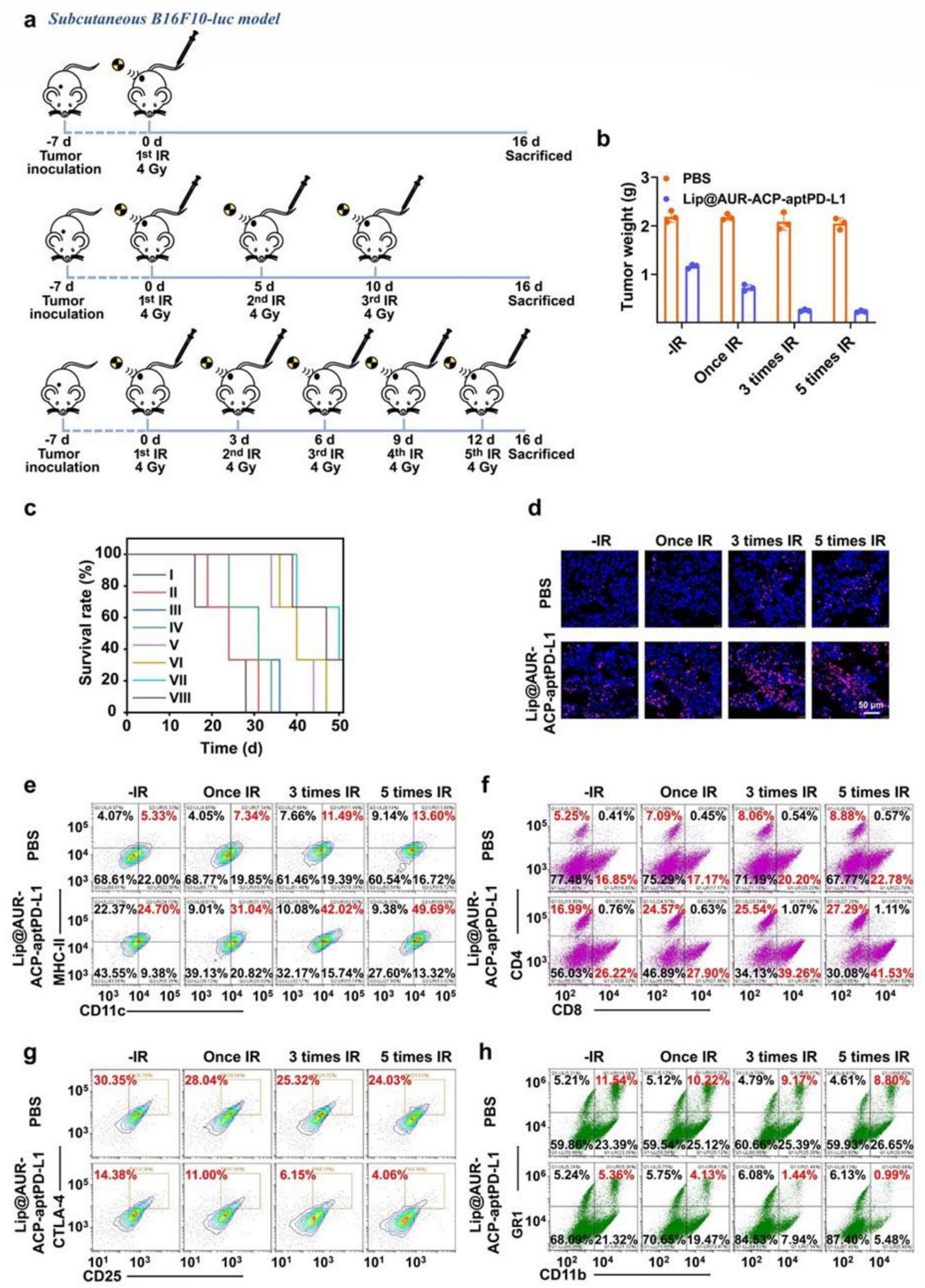

Supplementary Figure 34. Evaluation on the effect of IR fractionations on the therapeutic potency in vivo. (a) Schematic representation of the treatment protocol for B16F10-luc tumor-bearing mice with 4 Gy IR under different fractionation set-ups. (b)

Final tumor weight at the end of the incubation period. (c) Survival curve of B16F10 tumor-bearing mice after different treatments. I: PBS-IR, II: PBS+Once IR, III: PBS+3 times IR, IV: PBS+5 times IR, V: Lip@AUR-ACP-aptPD-L1-IR, VI: Lip@AUR-ACP-aptPD-L1+Once IR, VII: Lip@AUR-ACP-aptPD-L1+3 times IR, VIII: Lip@AUR-ACP-aptPD-L1+5 times IR. (d) TUNEL staining of B16F10 tumor tissue samples after different treatments with three mice per group. (e-h) Flow cytometry analysis on the infiltration of DCs (CD11c+MHC-II+), effector T cells (CD3+CD4+CD8+), Tregs (CD4+CD25+CTLA-4+) and MDSCs (CD11b+GR1+) in the B16F10 tumor tissues after different treatments with three mice per group. Data are presented as mean values ± SEM (n = 3 mice for panels b-c). Source data are provided as a Source Data file.

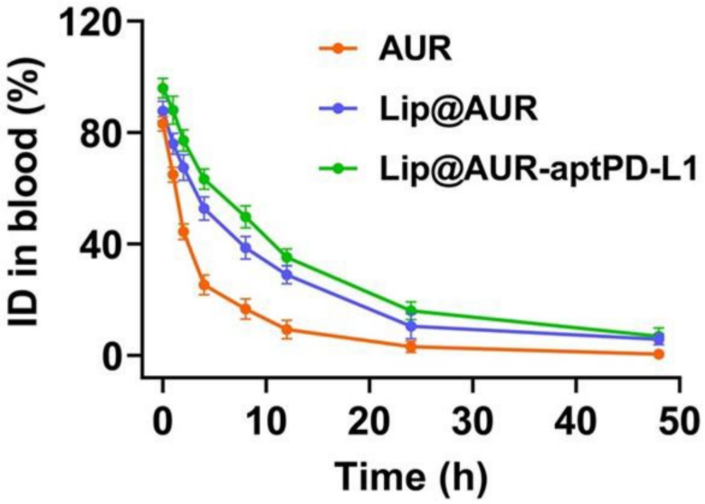

Supplementary Figure 35. Pharmacokinetic analysis in vivo. Blood circulation time of different samples in C57BL/6J mice. Data are presented as mean values ± SEM (n = 3 mice). Source data are provided as a Source Data file.

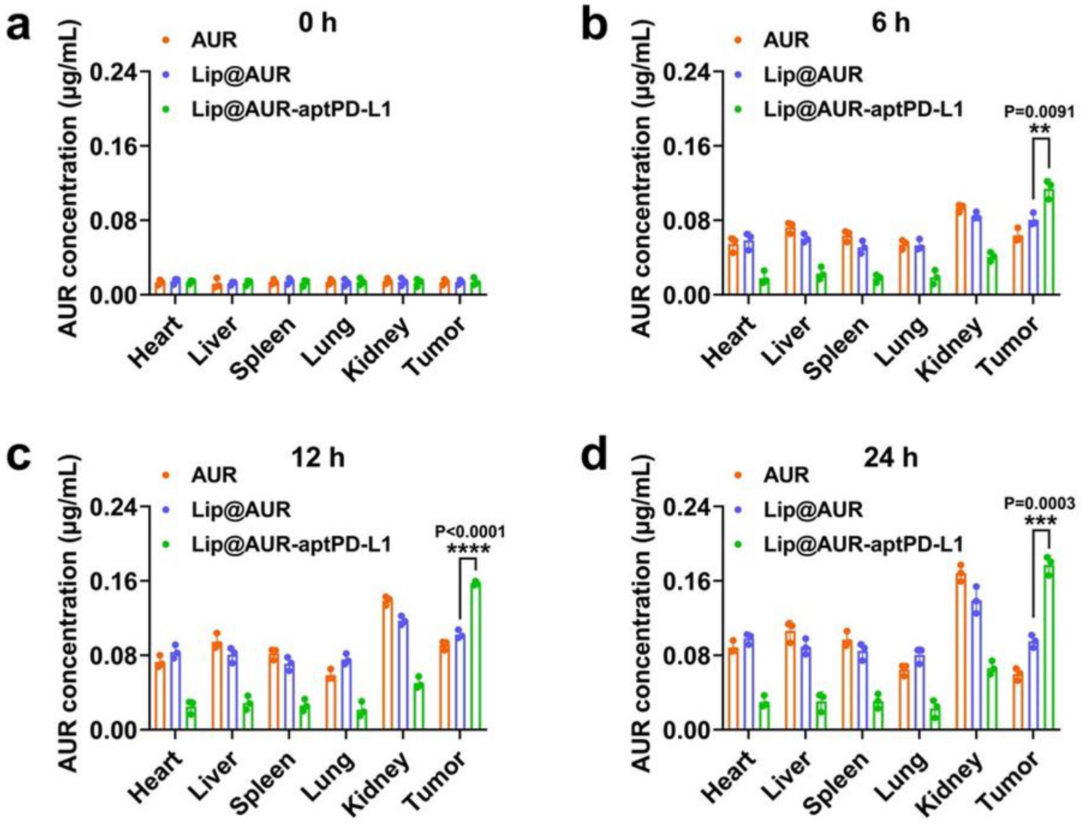

Supplementary Figure 36. Biodistribution analysis of the liposomes in vivo. ICP analysis of AUR deposition in major organs and tumors of B16F10 tumor-bearing mice after injection with AUR, Lip@AUR or Lip@AUR-aptPD-L1 at 0 h, 6 h, 12 h and 24 h. Data are presented as mean values ± SEM (n = 3 mice for panels a-d). Statistical analysis in panels b-d was carried out via one-way ANOVA method. * indicates significance at $p < 0.05$, ** indicates significance at $p < 0.01$, *** indicates significance at $p < 0.001$, **** indicates significance at $p < 0.0001$. Source data are provided as a Source Data file.

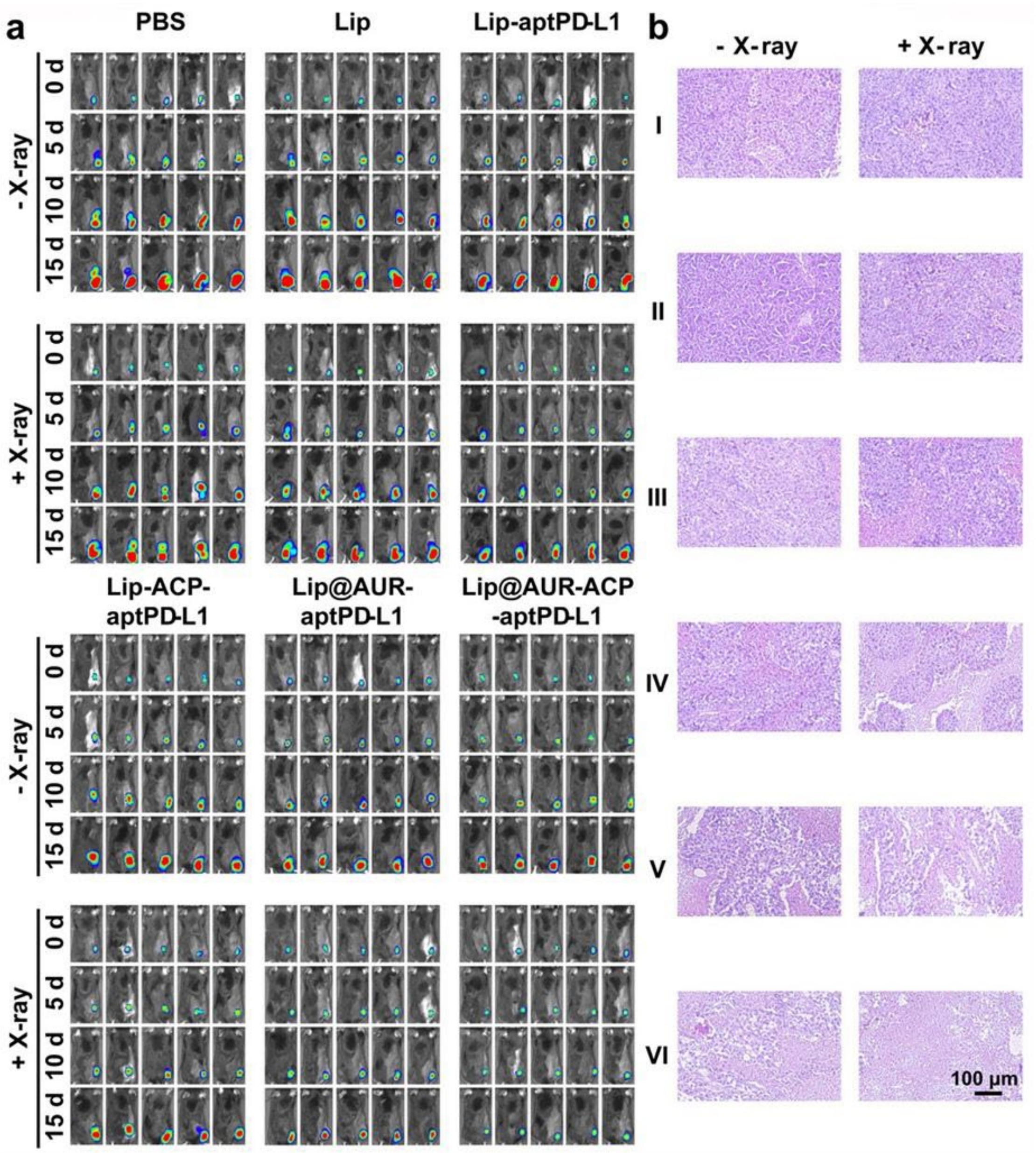

Supplementary Figure 37. Anti-tumor effects with different groups in vivo. (a) In vivo bioluminescence images of B16F10-Luc tumor-bearing mice during treatment with five mice per group. (b) Histological analysis on the treatment-induced anti-tumor effects according to H&E staining of B16F10 tumor tissues after different groups treatment with five mice per group. I: PBS, II: Lip, III: Lip-aptPD-L1, IV: Lip-ACP-aptPD-L1, V: Lip@AUR-aptPD-L1, VI: Lip@AUR-ACP-aptPD-L1.

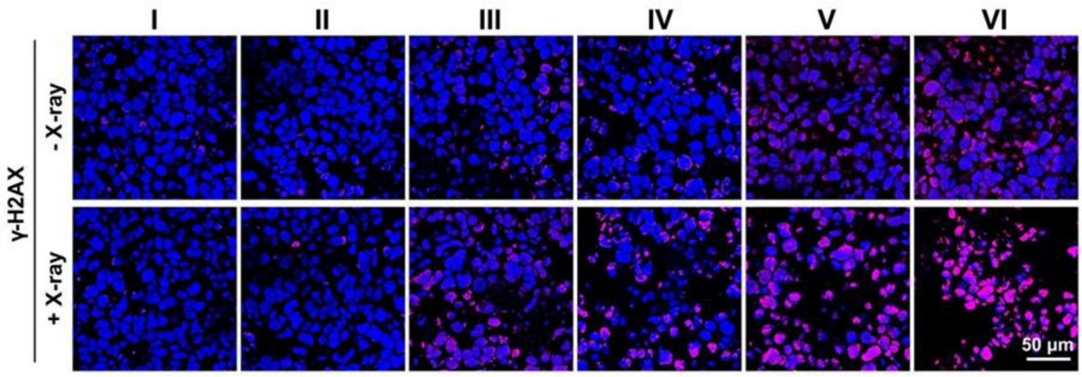

Supplementary Figure 38. Radiosensitization effect of the liposomes on B16F10 tumors. γ-H2AX immunofluorescence after different treatments with five mice per group. I: PBS, II: Lip, III: Lip-aptPD-L1, IV: Lip-ACP-aptPD-L1, V: Lip@AUR-aptPD-L1, VI: Lip@AUR-ACP-aptPD-L1. In vivo histological experiment was repeated three times independently with similar results.

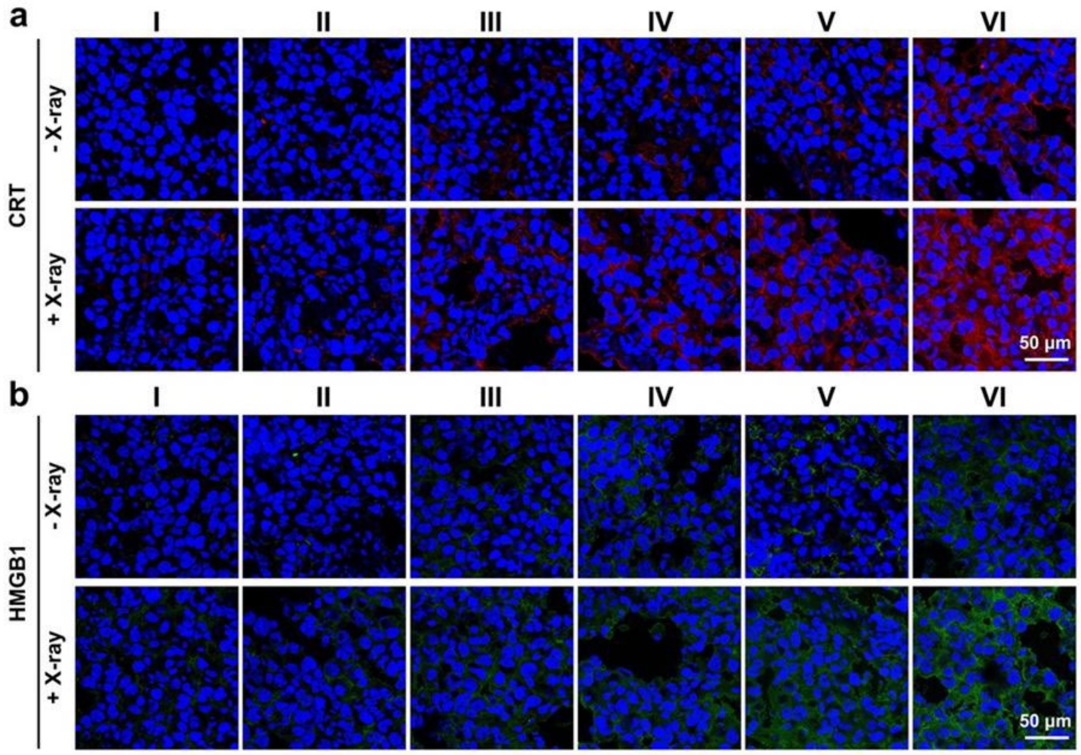

Supplementary Figure 39. Evaluation of treatment-induced ICD of B16F10 tumor tissues in vivo. CRT and HMGB1 immunofluorescence staining of the B16F10 tumors after different groups treatment with five mice per group. I: PBS, II: Lip, III: Lip-aptPD-L1, IV: Lip-ACP-aptPD-L1, V: Lip@AUR-aptPD-L1, VI: Lip@AUR-ACP-aptPD-L1. In vivo histological experiment in panel a-b were repeated three times independently with similar results.

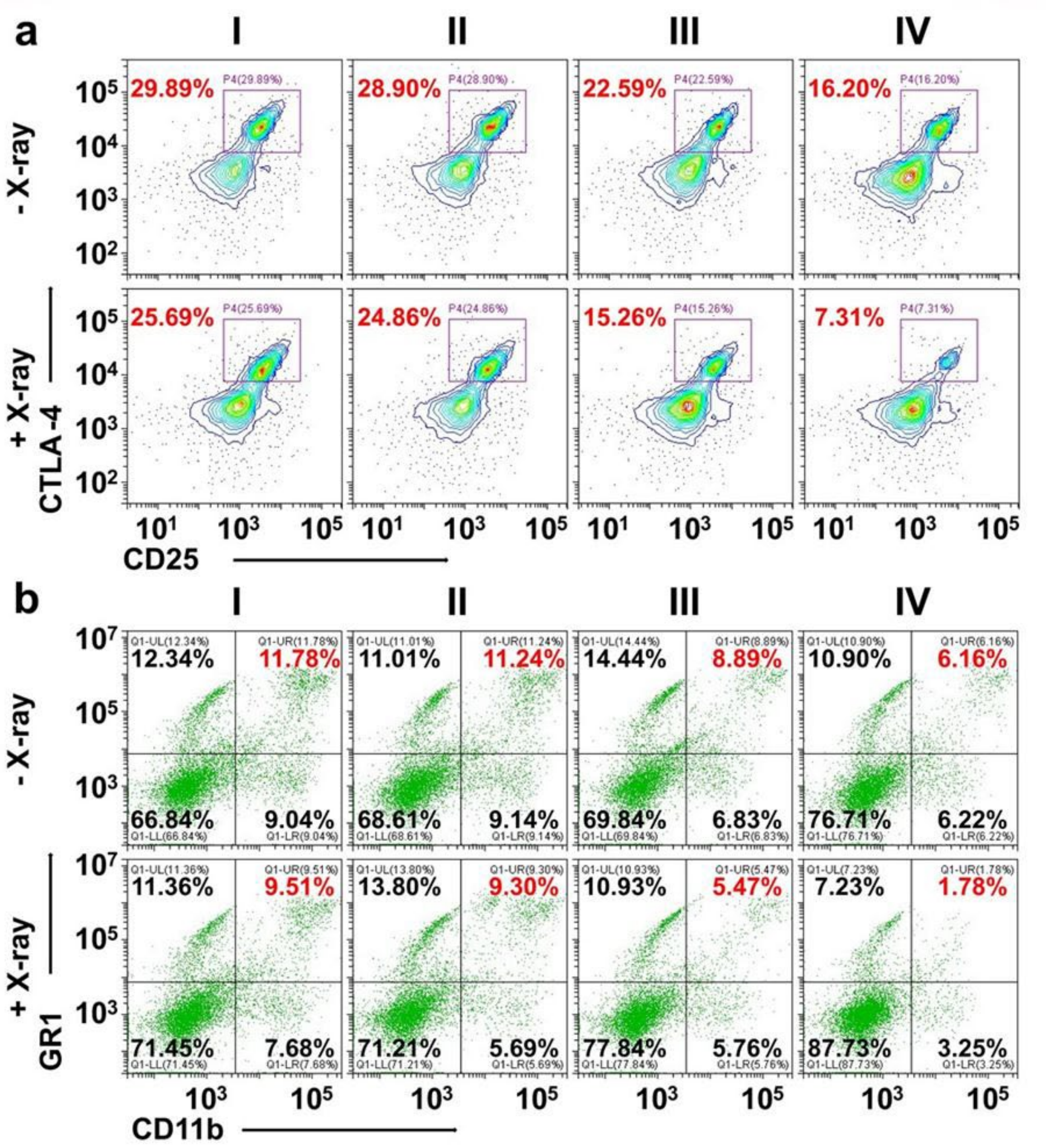

Supplementary Figure 40. In vivo evaluation on the tumor-residing immunosuppressor cell populations after various treatments. (a) Frequencies of Tregs (CD4+CD25+CTLA-4+) in B16F10 tumor tissues after different treatments with five mice per group. (b) Frequencies of MDSCs (CD11b+GR1+) in B16F10 tumor tissues after different treatments with five mice per group. I: PBS, II: Lip, III: Lip-@AUR, IV: Lip@AUR-aptPD-L1.

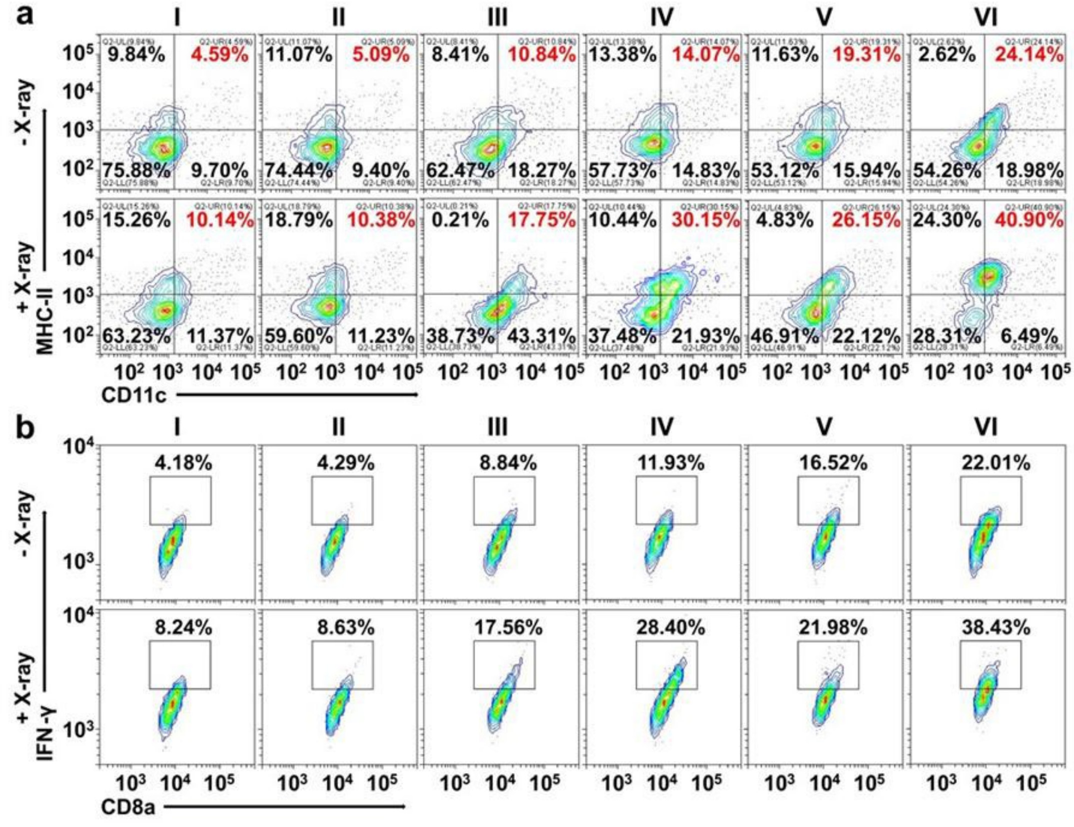

Supplementary Figure 41. Treatment-induced remodeling of B16F10 tumor immune microenvironment. (a) Frequencies of DCs (CD11c+MHC-II+) in B16F10 tumor tissues after different groups treatment with five mice per group. (b) Frequencies of effector CD8+T cells (CD8a+IFN-γ+) in B16F10 tumor tissues after different groups treatment with five mice per group. I: PBS, II: Lip, III: Lip-aptPD-L1, IV: Lip-ACP-aptPD-L1, V: Lip@AUR-aptPD-L1, VI: Lip@AUR-ACP-aptPD-L1.

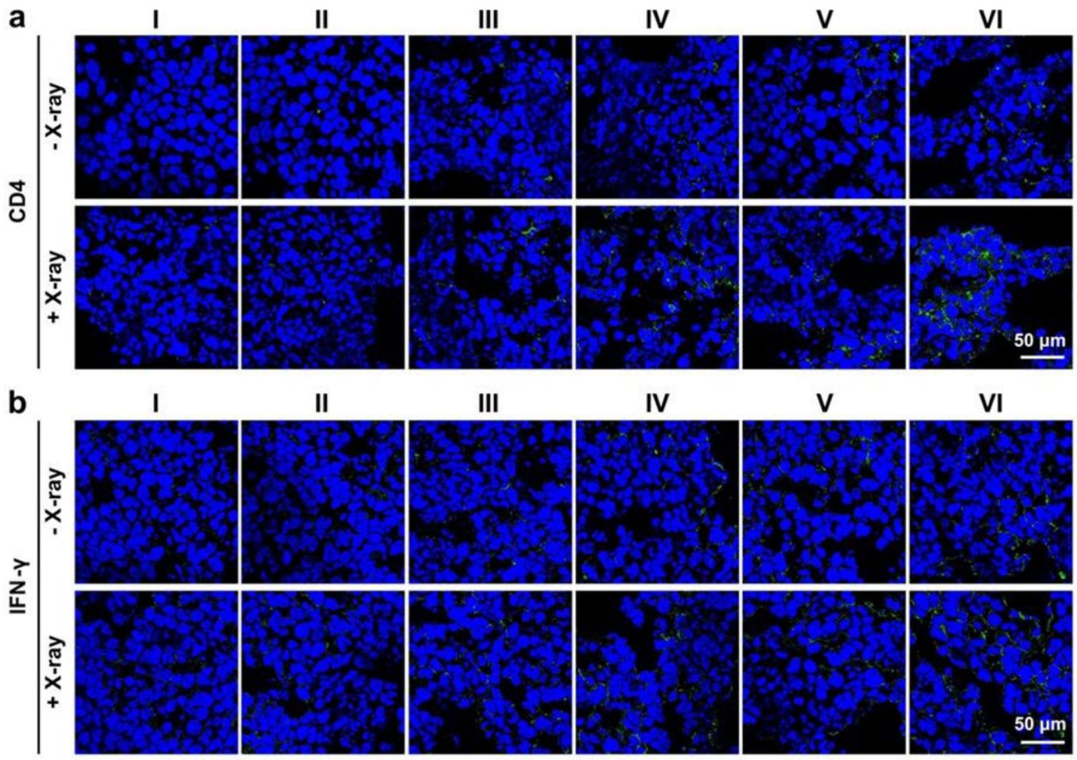

Supplementary Figure 42. T cell infiltration in B16F10 tumor tissues. (a) Immunofluorescence staining of CD4+T cells in B16F10 tumor tissues after different groups treatment with five mice per group. (b) Immunofluorescence staining of IFN-γ+ in B16F10 tumor tissues after different groups treatment with five mice per group. I: PBS, II: Lip, III: Lip-aptPD-L1, IV: Lip-ACP-aptPD-L1, V: Lip@AUR-aptPD-L1, VI: Lip@AUR-ACP-aptPD-L1. In vivo histological experiment in panel a-b were repeated three times independently with similar results.

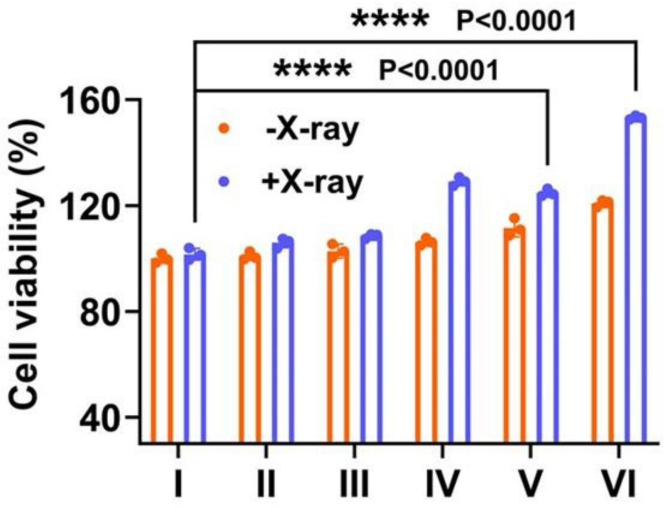

Supplementary Figure 43. Detection of CD3+T cell infiltration in B16F10 tumors treated with different materials by CCK8 assay. The group set-ups include I: PBS, II: Lip, III: Lip-aptPD-L1, IV: Lip-ACP-aptPD-L1, V: Lip@AUR-aptPD-L1, VI: Lip@AUR-ACP-aptPD-L1. Data are presented as mean values ± SEM (n = 3 mice). Statistical analysis was carried out via one-way ANOVA method. * indicates significance at $p < 0.05$, ** indicates significance at $p < 0.01$, *** indicates significance at $p < 0.001$, **** indicates significance at $p < 0.0001$. Source data are provided as a Source Data file.

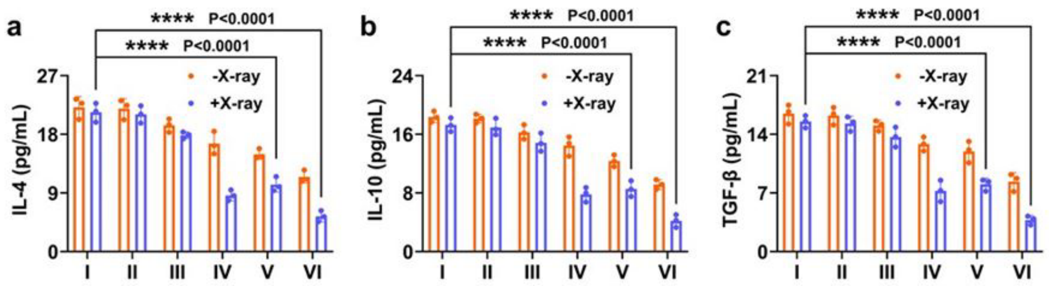

Supplementary Figure 44. Evaluation on the post-treatment secretion levels of anti-inflammatory factors in vivo. Secretion of various anti-inflammatory factors including (a) IL-4, (b) IL-10 and (c) TGF-β after different treatment in B16F10 tumor-bearing mice. I: PBS, II: Lip, III: Lip-aptPD-L1, IV: Lip-ACP-aptPD-L1, V: Lip@AUR-aptPD-L1, VI: Lip@AUR-ACP-aptPD-L1. Data are presented as mean values ± SEM (n = 3 mice for panels a-c). Statistical analysis in panels a-c was carried out via one-way ANOVA method. * indicates significance at $p < 0.05$, ** indicates significance at $p < 0.01$, *** indicates significance at $p < 0.001$, **** indicates significance at $p < 0.0001$. Source data are provided as a Source Data file.

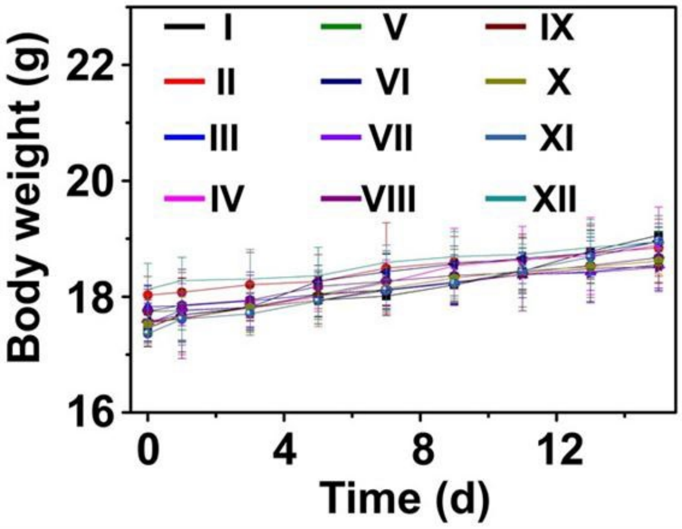

Supplementary Figure 45. Body weight analysis of the mouse models. Biocompatibility analysis by monitoring the body weight changes of B16F10 tumor-bearing mouse after different treatments. I: PBS, II: Lip, III: Lip-aptPD-L1, IV: Lip-ACP-aptPD-L1, V: Lip@AUR-aptPD-L1, VI: Lip@AUR-ACP-aptPD-L1, VII: PBS+IR, VIII: Lip+IR, IX: Lip-aptPD-L1+IR, X: Lip-ACP-aptPD-L1+IR, XI: Lip@AUR-aptPD-L1+IR, XII: Lip@AUR-ACP-aptPD-L1+IR. Data are presented as mean values ± SEM (n = 5 mice). Source data are provided as a Source Data file.

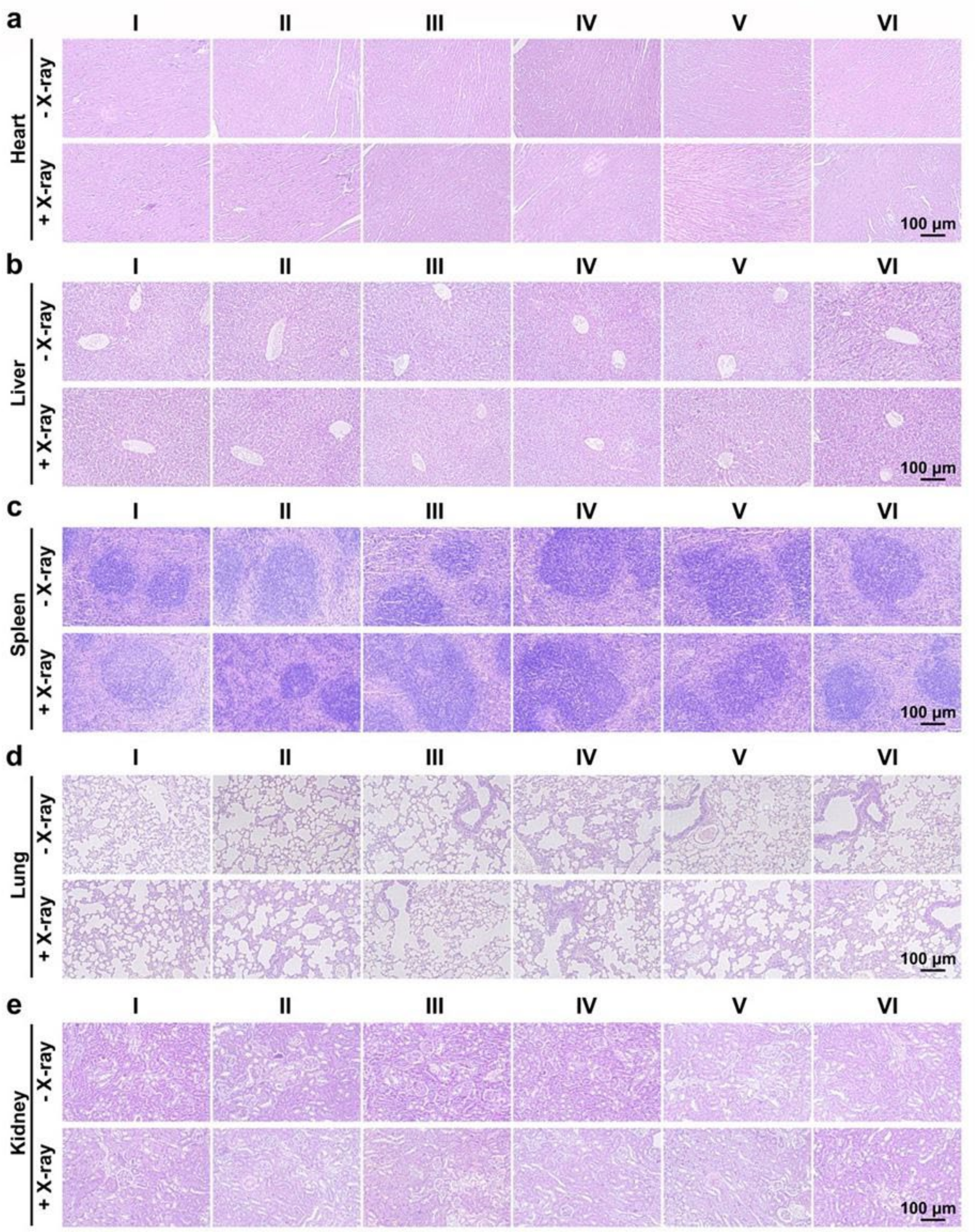

Supplementary Figure 46. Histopathological analysis on the biocompatibility of the liposome-augmented radio-immunotherapy in vivo. Histological analysis on the biocompatibility of the liposome-augmented radio-immunotherapy in vivo according to H&E staining of mouse major organs after different groups treatment with five mice per group. I: PBS, II: Lip, III: Lip-aptPD-L1, IV: Lip-ACP-aptPD-L1, V: Lip@AUR-

aptPD-L1, VI: Lip@AUR-ACP-aptPD-L1.

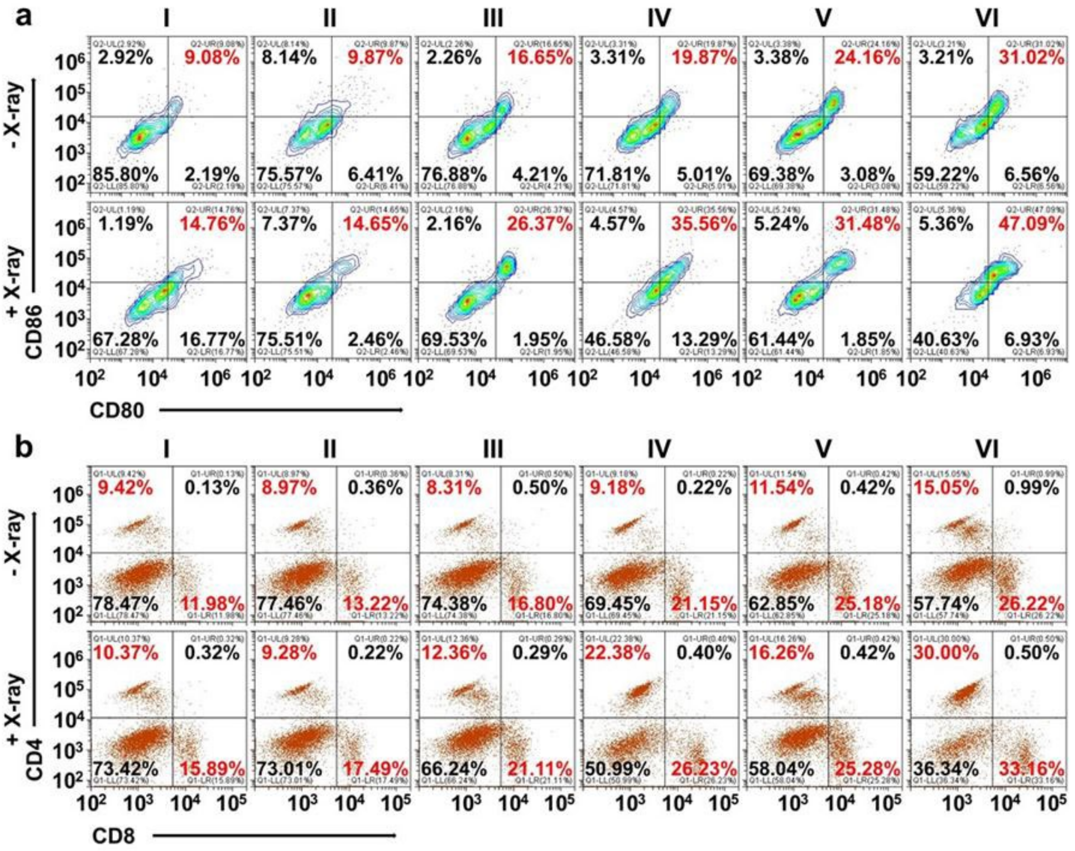

Supplementary Figure 47. Evaluation on treatment-evoked systemic immunity in the primary B16F10 tumor tissues. (a) Frequencies of DCs (CD11c+CD80+CD86+) in the primary B16F10 tumor tissues after different groups treatment with three mice per group. (b) effector T cells (CD3+CD4+CD8+) in the primary B16F10 tumor tissues after different groups treatment with three mice per group. I: PBS, II: Lip, III: Lip-aptPD-L1, IV: Lip-ACP-aptPD-L1, V: Lip@AUR-aptPD-L1, VI: Lip@AUR-ACP-aptPD-L1.

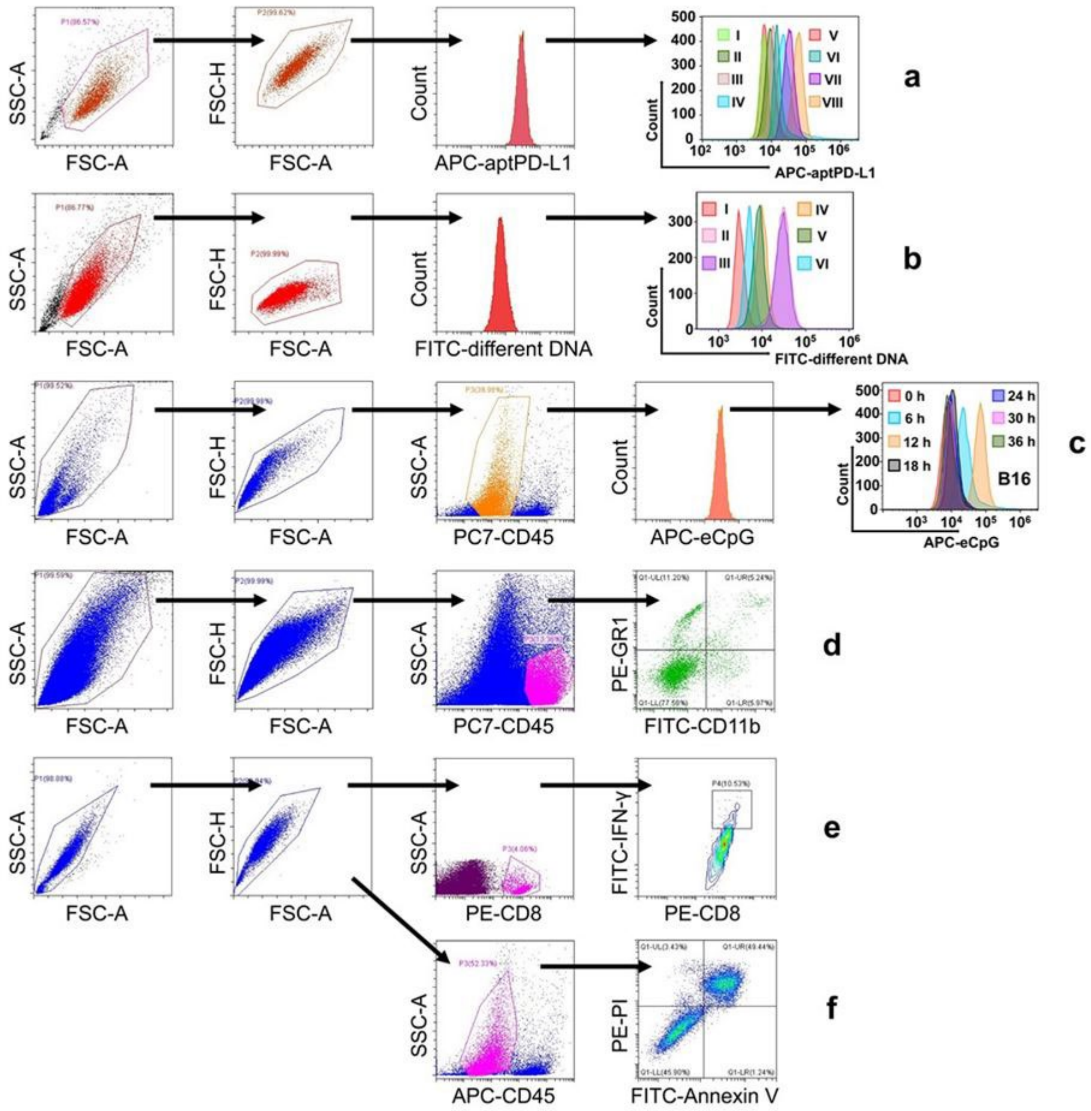

Supplementary Figure 48. Gating strategy for the FACS tests. a was applied for Supplementary Figure 9b; b was applied for Figure 4e; c was applied for Figure 4j; d was applied for Supplementary Figure 20b, Supplementary Figure 32h, Supplementary Figure 34h and Supplementary Figure 40b; e was applied for Supplementary Figure 25b and Supplementary Figure 41b; f was applied for Figure 5g, Supplementary Figure 26c and Supplementary Figure 27c.

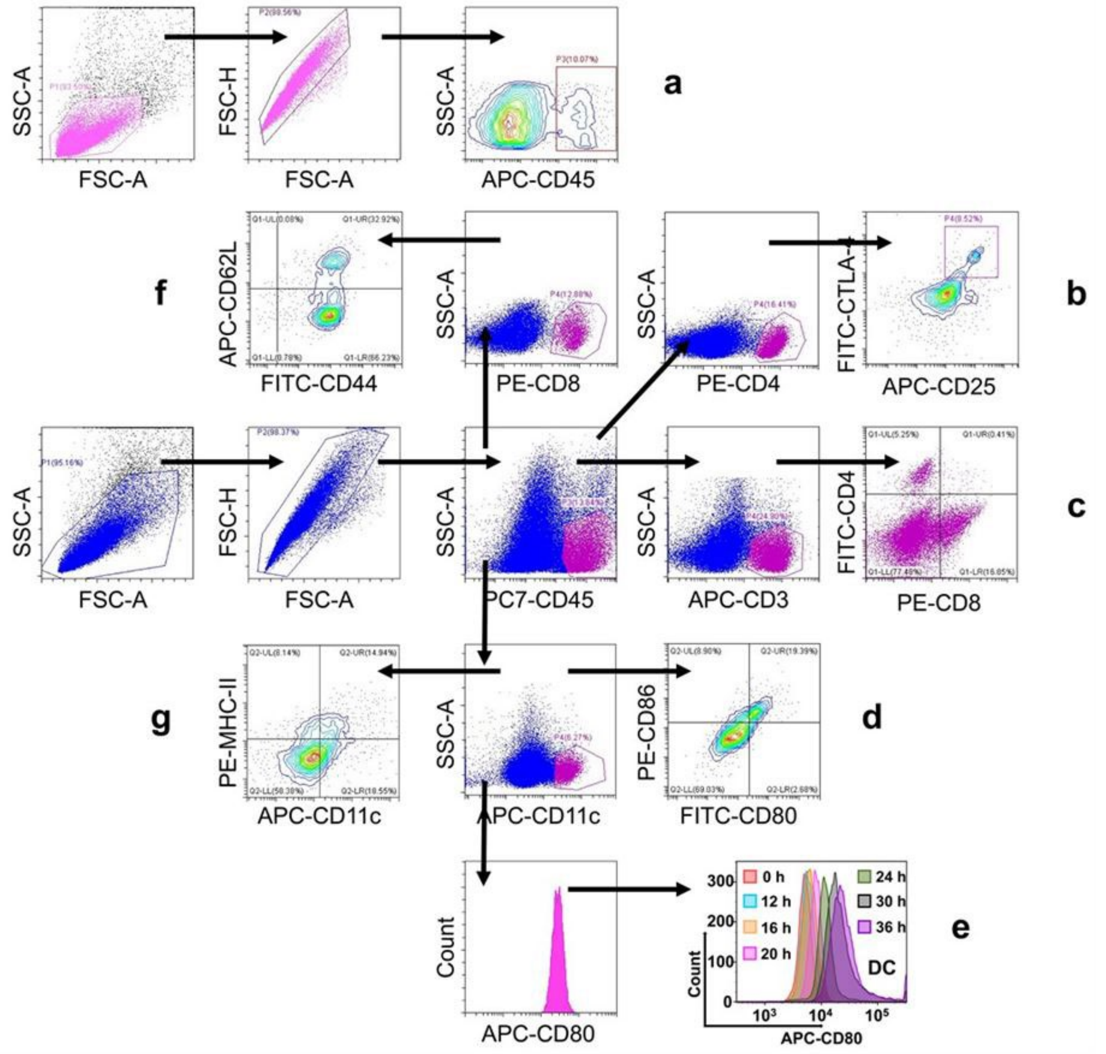

Supplementary Figure 49. Gating strategy for the FACS tests. a was applied for Figure 7a; b was applied for Supplementary Figure 20a, Supplementary Figure 32g, Supplementary Figure 34g and Supplementary Figure 40a; c was applied for Figure 5b, Figure 7c, Figure 8e, Supplementary Figure 21a, Supplementary Figure 26b, Supplementary Figure 27b, Supplementary Figure 32f, Supplementary Figure 34f and Supplementary Figure 47b; d was applied for Figure 4f, Figure 4k, Figure 4l, Figure 5a, Figure 7b, Figure 8d, Supplementary Figure 21b, Supplementary Figure 26a, Supplementary Figure 27a and Supplementary Figure 47a; e was applied for Figure 4m; f was applied for Figure 8f; g was applied for Supplementary Figure 25a,

Supplementary Figure 32e, Supplementary Figure 34e and Supplementary Figure 41a.

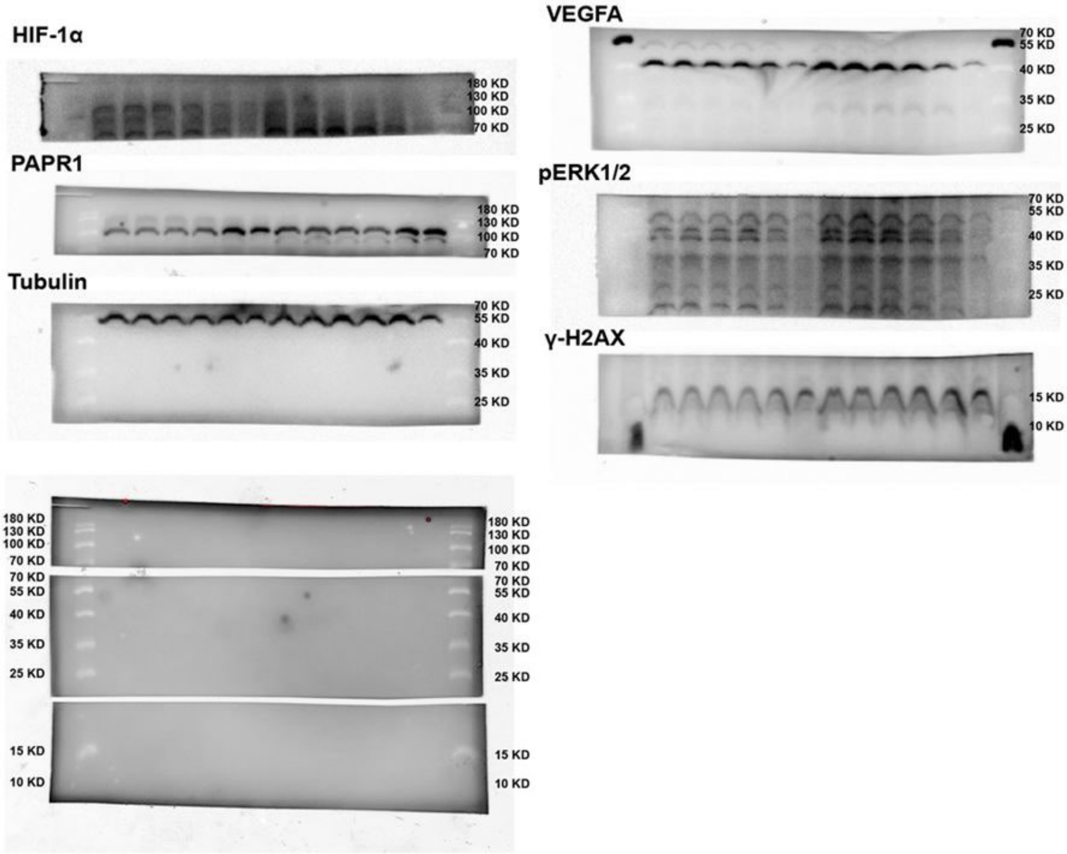

Supplementary Figure 50. Uncropped scans of western blot with marked molecular weight distributions for Supplementary Figure 18.
