# Supplementary material for: Programmable melanoma-targeted radio-immunotherapy via fusogenic liposomes functionalized with multivariate-gated aptamer assemblies
Source: Nat Commun. 2024 Jun 12;15:5035. doi: 10.1038/s41467-024-49482-9 (PMC11169524; doi:10.1038/s41467-024-49482-9)
Supplement: Supplementary file 3 — Reporting Summary [file 41467_2024_49482_MOESM3_ESM.pdf]

nature portfolio

Corresponding author(s): Menghuan Li, Zhong Luo, Fudi Wang

Last updated by author(s): Jan 5, 2024

Reporting Summary

Nature Portfolio wishes to improve the reproducibility of the work that we publish. This form provides structure for consistency and transparency in reporting. For further information on Nature Portfolio policies, see our Editorial Policies and the Editorial Policy Checklist.

Statistics

For all statistical analyses, confirm that the following items are present in the figure legend, table legend, main text, or Methods section.

n/a	Confirmed	
☐	☒	The exact sample size (*n*) for each experimental group/condition, given as a discrete number and unit of measurement
☐	☒	A statement on whether measurements were taken from distinct samples or whether the same sample was measured repeatedly
☐	☒	The statistical test(s) used AND whether they are one- or two-sided<br>*Only common tests should be described solely by name; describe more complex techniques in the Methods section.*
☐	☒	A description of all covariates tested
☐	☒	A description of any assumptions or corrections, such as tests of normality and adjustment for multiple comparisons
☐	☒	A full description of the statistical parameters including central tendency (e.g. means) or other basic estimates (e.g. regression coefficient) AND variation (e.g. standard deviation) or associated estimates of uncertainty (e.g. confidence intervals)
☐	☒	For null hypothesis testing, the test statistic (e.g. *F*, *t*, *r*) with confidence intervals, effect sizes, degrees of freedom and *P* value noted<br>*Give P values as exact values whenever suitable.*
☒	☐	For Bayesian analysis, information on the choice of priors and Markov chain Monte Carlo settings
☒	☐	For hierarchical and complex designs, identification of the appropriate level for tests and full reporting of outcomes
☐	☒	Estimates of effect sizes (e.g. Cohen's *d*, Pearson's *r*), indicating how they were calculated

Our web collection on statistics for biologists contains articles on many of the points above.

Software and code

Policy information about availability of computer code

Data collection	LAS AF Lite, CytExpert, FlowJo-V10, ChemDraw
Data analysis	All statistical data were processed in GraphPad Prism (version 9.5 for Windows) by Student's t-test and one-way ANOVA. All data was managed using Origin 2018 64Bit or GraphPad Prism (version 9.5 for Windows) . All figures are created by Adobe Illustrator 2020 and PowerPoint 2020.

For manuscripts utilizing custom algorithms or software that are central to the research but not yet described in published literature, software must be made available to editors and reviewers. We strongly encourage code deposition in a community repository (e.g. GitHub). See the Nature Portfolio guidelines for submitting code & software for further information.

Data

Policy information about availability of data

All manuscripts must include a data availability statement. This statement should provide the following information, where applicable:

- Accession codes, unique identifiers, or web links for publicly available datasets
- A description of any restrictions on data availability
- For clinical datasets or third party data, please ensure that the statement adheres to our policy

The generated sequencing data by Sangon (Shanghai, China) were deposited in https://www.ncbi.nlm.nih.gov/sra/PRJNA1100325, of which the accession code was PRJNA1100325. All remaining data can be found in the Article, Supplementary and Source data Files.

Research involving human participants, their data, or biological material

Policy information about studies with human participants or human data. See also policy information about sex, gender (identity/presentation), and sexual orientation and race, ethnicity and racism.

Reporting on sex and gender	n/a
Reporting on race, ethnicity, or other socially relevant groupings	n/a
Population characteristics	n/a
Recruitment	n/a
Ethics oversight	n/a

Note that full information on the approval of the study protocol must also be provided in the manuscript.

Field-specific reporting

Please select the one below that is the best fit for your research. If you are not sure, read the appropriate sections before making your selection.

☒ Life sciences ☐ Behavioural & social sciences ☐ Ecological, evolutionary & environmental sciences

For a reference copy of the document with all sections, see nature.com/documents/nr-reporting-summary-flat.pdf

Life sciences study design

All studies must disclose on these points even when the disclosure is negative.

Sample size	Sample size was determined based on the set-ups in previous reports to ensure adequate power (Nat Commun. 2022, 13(1), 5685).
Data exclusions	No data exclusion was performed.
Replication	All measurements were performed on three or more independent replicates from separate experiments.
Randomization	Samples and cells were randomly allocatad into groups. Mice with comparable age, weight and tumor sizes were randomly selected from the housing cages and then divided into experimental groups with no bias for further treatment.
Blinding	Investigators were blinded to group allocation during all experiments.

Reporting for specific materials, systems and methods

We require information from authors about some types of materials, experimental systems and methods used in many studies. Here, indicate whether each material, system or method listed is relevant to your study. If you are not sure if a list item applies to your research, read the appropriate section before selecting a response.

Materials & experimental systems

n/a	Involved in the study	
☐	☒	Antibodies
☐	☒	Eukaryotic cell lines
☒	☐	Palaeontology and archaeology
☐	☒	Animals and other organisms
☒	☐	Clinical data
☒	☐	Dual use research of concern
☒	☐	Plants

Methods

n/a	Involved in the study	
☒	☐	ChIP-seq
☐	☒	Flow cytometry
☒	☐	MRI-based neuroimaging

Antibodies

Antibodies used	PC7-anti-mouse CD45 antibody, APC-anti-mouse CD45 antibody, APC-anti-mouse CD3 antibody, APC-anti-mouse CD11c antibody, PE-anti-mouse CD8a antibody, PE-anti-mouse CD4 antibody, PE-anti-mouse CD86 antibody, FITC-anti-mouse IFN-γ antibody, FITC-anti-mouse CD44 antibody, PE-anti-mouse MHC-II antibody, FITC-anti-mouse CD4 antibody, PE-anti-mouse CD11c antibody, PE-anti-

mouse GR1 antibody, FITC-anti-mouse CD11b antibody, FITC-anti-mouse CD80 antibody, APC-anti-mouse CD25 antibody, APC-anti-mouse CD62L antibody, APC-anti-mouse CD80 antibody, FITC-anti-mouse PD-L1 antibody were purchased from elabscience (Wuhan, China), of which the catalog numbers were E-AB-F1136G, E-AB-F1136E, E-AB-F1013E, F-AB-F0991E, E-AB-F1104D, E-AB-F1097D, E-AB-F0994D, E-AB-F1101C, E-AB-F1100C, E-AB-F0990D, E-AB-F1097C, E-AB-F0991D, E-AB-F1120D, E-AB-F1081C, EAB-F0992C, E-AB-F1102E, E-AB-F1011E, E-AB-F0992E, E-AB-F1132C respectively. The dilution ratios of above-mentioned antibodies were 1:50.
FITC-anti-CTLA-4 antibody, anti-mouse Calretinin antibody, anti-mouse HMGB1 antibody and anti-PD-L1 antibody were purchased from Abcam (Shanghai, China), of which the catalog numbers were ab24935 (1:100), ab92341 (1:200), ab79823 (1:200) and ab213480 (1:200).
anti-mouse β-Tubulin antibody was purchased from Abmart (Shanghai, China), of which the catalog number was M30109S (1:1000).
anti-mouse VEGFA antibody, anti-mouse HIF-1α antibody, anti-mouse pERK1/2 antibody, anti-mouse PARP1 antibody, anti-mouse γH2AX antibody were purchased from proteintech (Wuhan, China), of which the catalog numbers were 19003-1-AP (1:500), 66730-1-Ig (1:500), 28733-1-AP (1:500), 66520-1-Ig (1:500), 10856-1-AP (1:500), respectively.

Validation

PC7-anti-CD45 antibody https://www.elabscience.cn/p-pe_cyanine5_anti_mouse_cd45_antibody_30_f11_-172697.html
APC-anti-CD45 antibody https://www.elabscience.cn/p-apc_anti_mouse_cd45_antibody_30_f11_-172695.html
APC-anti-CD3 antibody https://www.elabscience.cn/p-apc_anti_mouse_cd3_antibody_17a2_-133030.html
APC-anti-CD11c antibody https://www.elabscience.cn/p-apc_anti_mouse_cd11c_antibody_n418_-132974.html
PE-anti-CD8a antibody https://www.elabscience.cn/p-pe_anti_mouse_cd8a_antibody_53_6.7_-133053.html
PE-anti-CD4 antibody https://u2.elabscience.cn/p-pe_anti_mouse_cd4_antibody_gk1.5_-133045.html
PE-anti-CD86 antibody https://u2.elabscience.cn/p-pe_anti_mouse_cd86_antibody_gl_1_-172246.html
FITC-anti-IFN-γ antibody https://www.elabscience.cn/p-fitc_anti_mouse_ifn_gamma_antibody_xmg1.2_-172590.html
FITC-anti-CD44 antibody https://www.elabscience.cn/p-fitc_anti_human_mouse_cd44_antibody_im7_-176555.html
PE-anti-MHC-II antibody https://www.elabscience.cn/p-pe_anti_mouse_mhc_ii_i_a_i_e_antibody_m5_114_-132965.html
FITC-anti-CD4 antibody https://u2.elabscience.cn/p-fitc_anti_mouse_cd4_antibody_gk1.5_-133044.html
PE-anti-CD11c antibody https://www.elabscience.cn/p-pe_anti_mouse_cd11c_antibody_n418-e_ab_f0991d
PE-anti-GR1 antibody https://www.elabscience.cn/p-pe_anti_mouse_ly_6g_ly_6c_gr_1_antibody_rb6_8c5_-150590.html
FITC-anti-CD11b antibody https://www.elabscience.cn/p-fitc_anti_mouse_human_cd11b_antibody_m1_70_-133068.html
FITC-anti-CD80 antibody https://u2.elabscience.cn/p-fitc_anti_mouse_cd80_antibody_16_10a1_-172238.html
APC-anti-CD25 antibody https://www.elabscience.cn/p-apc_anti_mouse_cd25_antibody_pc_61.5.3_-134626.html
APC-anti-CD62L antibody https://www.elabscience.cn/p-apc_anti_mouse_cd62l_antibody_mel14_-172281.html
APC-anti-CD80 antibody https://u2.elabscience.cn/p-apc_anti_mouse_cd80_antibody_16_10a1_-134498.html
FITC-anti-CTLA-4 antibody https://www.abcam.cn/products/primary-antibodies/fitc-ctla4-antibody-1b8-ab24935.html
anti-VEGFA antibody https://www.ptgcn.com/products/VEGFA-Antibody-19003-1-AP.htm
anti-HIF-1α antibody https://www.ptgcn.com/products/HIF1a-Antibody-66730-1-Ig.htm
anti-pERK1/2 antibody https://www.ptgcn.com/products/ERK1-2-phospho-Thr202-Tyr204-Antibody-28733-1-AP.htm
anti-PARP1 antibody https://www.ptgcn.com/products/PARP1-Antibody-66520-1-Ig.htm
anti-γH2AX antibody https://www.ptgcn.com/products/H2AFX-Antibody-10856-1-AP.htm
anti-β-Tubulin antibody http://www.ab-mart.com.cn/page.aspx?node=%2059%20&id=%20992
anti-Calretinin antibody https://www.abcam.cn/products/primary-antibodies/calretinin-antibody-ep1798-ab92341.html
anti-HMGB1 antibody https://www.abcam.cn/products/primary-antibodies/hmgb1-antibody-epr3507-ab79823.html
anti-PD-L1 antibody https://www.abcam.cn/products/primary-antibodies/pd-l1-antibody-epr20529-ab213480.html
FITC-anti-PD-L1 antibody https://www.elabscience.cn/p-fitc_anti_mouse_cd274_pd_l1_antibody_10f_9g2-e_ab_f1132c

Eukaryotic cell lines

Policy information about cell lines and Sex and Gender in Research

Cell line source(s)

B16F10, NIH3T3, B16F10-luc cell lines were purchased from Shanghai Zeye Biotechnology Co., Ltd. with the catalog number of ZY-C6002M, ZY-C6050M, ZY-C6002M-L, respectively.

Authentication

These cell lines were authenticated by the supplier using STR analysis.

Mycoplasma contamination

No contamination was detected by the supplier using Hoechst DNA stain method, agar culture method and PCR-based assay.

Commonly misidentified lines
(See ICLAC register)

No commonly misidentified cell lines were used in the study.

Animals and other research organisms

Policy information about studies involving animals; ARRIVE guidelines recommended for reporting animal research, and Sex and Gender in Research

Laboratory animals

C57BL/6J (female, 6-week-old) were provided by the Second Affiliated Hospital of the Army Medical University (Xinqiao Hospital) with License No. SCXK (Chongqing) 2022-0011 of Laboratory Animal Production and all mice were kept in the animal house of Xinqiao Hospital and housed in cages with five mice per cage and kept on in a regular 12 h light: 12 h dark cycle (9:00–21:00; 21:00–9:00). The temperature was 22 ± 1 degree Celsius and humidity was 40%-68%. All animal tests have been reviewed and approved by the Laboratory Animal Welfare and Ethics Committee of the Army Medical University with License No. SYXK (Chongqing) 2022-0018 of Laboratory Animal Facility, which were carried out following the Animal Management Rules of the Ministry of Health of the People's Republic of China.

Wild animals

No wild animals were used in the study.

Reporting on sex

Female mice were used in this study.

Field-collected samples	No field collected samples were used in the study.
Ethics oversight	All animal tests have been reviewed and approved by the Laboratory Animal Welfare and Ethics Committee of the Army Medical University, which carried out following the Animal Management Rules of the Ministry of Health of the People's Republic of China. According to the national and institutional guidelines, the maximum tumor size allowed was 2000 mm3, and mice were euthanized when the tumor burden exceeded the threshold.

Note that full information on the approval of the study protocol must also be provided in the manuscript.

Flow Cytometry

Plots

Confirm that:

☒ The axis labels state the marker and fluorochrome used (e.g. CD4-FITC).

☒ The axis scales are clearly visible. Include numbers along axes only for bottom left plot of group (a 'group' is an analysis of identical markers).

☒ All plots are contour plots with outliers or pseudocolor plots.

☒ A numerical value for number of cells or percentage (with statistics) is provided.

Methodology

Sample preparation

Flow cytometric analysis on the receptor binding effect of aptPD-L1 and eCpG: B16F10 cells were mixed with splenocytes at a ratio of 1:10 and transferred to a 1.5 mL centrifuge tube. 170 nM aptPD-L1FAM and 360 nM eCpGFAM were added and incubated for 30 min with 5% BSA, followed by the addition of the corresponding antibodies (column 2 and 12, Supplementary Table 2) for 30 min incubation after washing with PBS. Flow cytometry was used to detect the binding status of aptPD-L1FAM and eCpGFAM.
Impact of IR treatment on B16F10-intrinsic PD-L1 expression: After 12 h treatment with 20 μg·mL-1 AUR, B16F10 cells were treated with different radiation doses including 0 Gy, 2 Gy, 4 Gy and 8 Gy. After 30 h incubation, the above samples were fixed with 4% paraformaldehyde for 30 min, followed by the addition of anti-PD-L1 antibody (column 28, Supplementary Table 2) and incubated at 4? overnight. Afterwards, FAM-labeled fluorescent secondary antibody was added and the cell samples were further incubated at room temperature for 2 h. The secondary antibody was removed and the cell nuclei were stained with DAPI for 10 min after washing with PBS. After cleaning, the immunofluorescence of PD-L1 was detected by confocal laser microscopy.
After 12 h treatment with 20 μg·mL-1 AUR, B16F10 cells were treated with different radiation doses including 0 Gy, 2 Gy, 4 Gy and 8 Gy. After 30 h incubation, the cells were detached with trypsin and sealed with 5% BSA for 30 min. The cells were incubated with FITC-anti-PD-L1 antibody (column 29, Supplementary Table 2) at 4? for 30 min. After cleaning, the PD-L1 expression was detected by flow cytometry.
Observation of eCpG release in vivo: B16F10-bearing C57BL/6J mouse models were treated with Lip@AUR-ACCy5P-aptPD-L1 (2 mg·kg-1) by intravenous injection. The time point of administration was defined as 0 h, while 4 Gy IR treatment was applied at 12 h post intravenous injection. The tumors were extracted at the time points of 0, 6, 12, 18, 24, 30 and 36 h, pulverized and filtered. The above cells were mixed with 5 mL red blood cell lysis buffer and stood for 10 min, and then centrifuged at 666.7 × g for 5 min. Subsequently, the cells were stained with the corresponding antibodies (column 1, Supplementary Table 2) for 30 min and washed with PBS. Finally, the Cy5 fluorescence of eCpG was quantified by flow cytometry.
Analysis of eCpG-mediated stimulation of DC maturation in vitro and in vivo: Lip@AUR-ACP-aptPD-L1 was added into the co-incubation system of B16F10 and mouse splenocytes and incubated for 12 h until 4 Gy IR was applied. At 12, 16, 20, 24, 30 or 36 h post liposome administration, the above cells were collected and added with the corresponding antibodies (column 1, 4, 7 and 15, Supplementary Table 2) for 30 min. After washing with PBS, the DC maturation status was detected by flow cytometry.
B16F10-bearing C57BL/6J mouse models were treated with Lip@AUR-ACP-aptPD-L1 (2 mg·kg-1) by intravenous injection. The time point of administration was defined as 0 h, while 4 Gy IR treatment was applied at 12 h post intravenous injection. The tumors were extracted at the time points of 0, 6, 12, 18, 24, 30 and 36 h, pulverized and filtered. The above cells were mixed with 5 mL red blood cell lysis buffer and stood for 10 min, and then centrifuged at 666.7 × g for 5 min. Subsequently, the cells were stained with the corresponding antibodies (column 1, 4, 7 and 15, Supplementary Table 2) for 30 min. After washing with PBS, the DC maturation status was detected by flow cytometry.
Evaluation on the impact of VEGF on anti-tumor immunity: B16F10 cells were inoculated into the 12-well plate at the concentration of 1×105 per well. When the cell confluence reached 80%, the cells were treated with PBS, Lip, Lip@AUR or Lip@AUR-aptPD-L1, the upper chamber is placed into 12-well plate. Splenocytes were added into the upper chamber with B16F10: splenocyte ratio of 1:10. After 12 h incubation, the IR groups were treated with 4 Gy IR. After 30 h incubation, the cells in the upper chamber were discarded and the bottom chamber supernatant was collected. After centrifugation at 666.7 × g for 5 min, 200 μL PBS was added to each tube to resuspend the spleen immune cells. The corresponding antibodies (column 1, 6, 13, 14, 16 and 19, Supplementary Table 2) were added into each tube. Finally, the infiltration of Tregs or MDSCs in the bottom chamber was detected by flow cytometry.
Alternatively, the recovered cell samples in the bottom chamber were treated with the corresponding antibodies (column 1, 3, 4, 5, 7, 11 and 15, Supplementary Table 2). Finally, the infiltration of effector T cells or DCs was detected by flow cytometry.
The B16F10 tumor-bearing mouse model was constructed and treated with PBS, Lip, Lip@AUR or Lip@AUR-aptPD-L1 (2mg·kg-1) by intravenous injection and treated with 4 Gy IR after 12 h post intravenous injection. After 30 h post intravenous injection, the tumors were collected from each group after treatment and pulverized to collect various cell populations. 200 μL PBS was added to each tube to suspend tumor cells. The corresponding antibodies (column 1, 6, 13, 14, 16 and 19, Supplementary Table 2) were added into each tube. Finally, the infiltration of Tregs or MDSCs in tumor tissues was

detected by flow cytometry.
Evaluation of treatment-induced immunoactivation in vitro: Splenocytes of C57BL/6J mice were extracted and DCs were sorted out according to the above method. B16F10 cells were inoculated into 12-well plates with the initial cell density of 1×105 cells/well. When the cell confluence reached 80%, mouse DCs were added into 12-well plates and co-cultured with B16F10 cells at a ratio of B16F10: DC=1:10. After 12 h treatment with PBS, Lip, Lip-aptPD-L1, Lip-ACP-aptPD-L1, Lip@AUR-aptPD-L1 or Lip@AUR-ACP-aptPD-L1, the IR groups were treated with 4 Gy IR. After 30 h incubation, DCs were collected via centrifugation. DCs was resuspended with 200 µL PBS and then incubated with the corresponding antibodies (column 1, 4, 7 and 15, Supplementary Table 2) for 30 min. Finally, the treatment-induced stimulation effect on DCs maturation in each group was detected by flow cytometry.
After B16F10 cells were inoculated into the 12-well plate through the procedure described above, mouse splenocytes were added into the 12-well plate and co-cultured with B16F10 cells at the B16F10: splenocyte ratio of 1:10. After 12 h treatment with PBS, Lip, Lip-aptPD-L1, Lip-ACP-aptPD-L1, Lip@AUR-aptPD-L1 or Lip@AUR-ACP-aptPD-L1, the IR groups were treated with 4 Gy IR. After 30 h incubation, spleen cells and supernatants were collected for later use. Here the spleen cells were suspended with 200 µL PBS, then the corresponding antibodies (column 1, 3, 5, 8 and 11, Supplementary Table 2) were added to each tube. Finally, the activation status of T cells in each group was detected by flow cytometry.
Detection of tumor cell apoptosis: The co-incubation system of B16F10 cells and mouse splenocytes were treated with PBS, Lip, Lip-aptPD-L1, Lip-ACP-aptPD-L1, Lip@AUR-aptPD-L1 or Lip@AUR-ACP-aptPD-L1 for 12 h. The IR groups were treated with 4 Gy IR. After 30 h incubation, all cells were collected and suspended with 200 µL FITC bonding solution at 37? for 30 min, then followed by PI dye solution for 10 min. After extensive staining, the corresponding antibodies (column 2, Supplementary Table 2) were added to each tube, then apoptosis of tumor cells under different treatments was detected by flow cytometry.

Instrument

Flow cytometry (CytoFLEX, Beckman Coulter)

Software

FlowJo-V10 and CytExpert

Cell population abundance

The cells stained with different markers were filtered using 300 screen mesh cell strainer. 10000 cells were extracted from individual samples for flow cytometry analysis.

Gating strategy

Living cells are first gated on the basis of their scattering properties using forward (FSC) and side scatter (SSC). Adhered particles and impurities were removed via FSCA/FSCH approach. Immune cells were gated via APC/SSCA approach to differentiate T cells. Activation status of T cells was determined by CD4/CD8 or CD8/IFN-γ expressions.

☒ Tick this box to confirm that a figure exemplifying the gating strategy is provided in the Supplementary Information.
